# Aerobic radical multifunctionalization of alkenes using *tert*-butyl nitrite and water

**DOI:** 10.3762/bjoc.9.196

**Published:** 2013-08-20

**Authors:** Daisuke Hirose, Tsuyoshi Taniguchi

**Affiliations:** 1 Graduate School of Natural Science and Technology, Kanazawa University, Kakuma-machi, Kanazawa 920-1192, Japan,; 2 School of Pharmaceutical Sciences, Institute of Medical, Pharmaceutical and Health Sciences, Kanazawa University, Kakuma-machi, Kanazawa 920-1192, Japan

**Keywords:** C−H oxidation, free radical, nitration, oxygen, radicals, water

## Abstract

Water induces a change in the product of radical multifunctionalization reactions of aliphatic alkenes involving an sp^3^ C–H functionalization by an 1,5-hydrogen shift using *tert*-butyl nitrite and molecular oxygen. The reaction without water, reported previously, gives nitrated γ-lactols, whereas the reaction in the presence of water produces 4-hydroxy-5-nitropentyl nitrate or 4-hydroxy-3-nitropentyl nitrate derivatives.

## Introduction

**Scheme 1:**
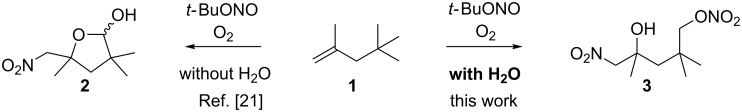
The effect of water in radical multifunctionalization reactions.

Multifunctionalization reactions of simple organic molecules are useful methods because they can provide a shortcut to desired products. Various methods used widely, such as organometallic reactions, cycloaddition reactions and multicomponent reactions, are utilized for multifunctionalization [1–5]. If direct functionalization reactions of inactivated bonds, such as simple multiple bonds or C–H bonds, are combined with multifunctionalization processes, they would become more efficient synthetic methods [6–9]. In this regard, radical reactions are capable of realizing multifunctionalization of inactivated organic molecules due to their high reactivity. Addition reactions of radical species to multiple bonds in the presence of appropriate trapping reagents give double functionalized compounds [10–11]. In addition, a radical methodology is a powerful tool for sp^3^ C–H functionalization [12–16]. It is frequently achieved by intramolecular radical hydrogen transfer reactions. An 1,5-hydrogen shift is the most favourable process [17], and useful methods such as the Barton reaction and the Hofmann–Löffler–Freytag reaction have been reported [18–20]. Recently, we reported a novel radical multifunctionalization reaction of aliphatic alkenes using *tert*-butyl nitrite (*t-*BuONO) and oxygen [21]. In this reaction, three positions including an unreactive sp^3^ C–H bond are functionalized in alkenes to produce nitrated γ-lactols in one step. For instance, treatment of alkene **1** with 5 equivalents of *t-*BuONO in a dried solvent under an oxygen atmosphere directly gave γ-lactol **2** (Scheme 1). On the other hand, we found in the course of this study that addition of water altered the products to a non-cyclic functionalized compound **3** (Scheme 1). In this letter, we report water-induced multifunctionalization reactions of alkenes by aerobic radical oxynitration.

## Results and Discussion

Our investigation started with the screening of solvents in the reaction of alkene **1** with *t*-BuONO (3 equiv) and oxygen in the presence of water (3 equiv). Although dimethyl sulfoxide (DMSO) was the best solvent in the reaction without water to obtain γ-lactol **2** [21], it worked inefficiently in the presence of water, and triple functionalized product **3** was obtained in low yield along with a large amount of oxynitration product **4** (Table 1, entry 1). The use of other polar solvents such as *N*,*N*-dimethylformamide (DMF) and tetrahydrofuran (THF) also did not give better results (Table 1, entries 2 and 3), whereas the use of dichloromethane (CH_2_Cl_2_) improved the ratio of **3** and **4** (ca. 1:2), though the isolated yield of **3** was still low (entry 4). Encouraged by this observation, we tested the reaction in pentane and obtained an improved ratio (ca. 1:1) and improved yield (26%) of **3** (entry 5). Eventually, the reaction with an excess of water (30 equiv) and with a longer reaction time (12 h) somewhat improved the yield (33%) of **3**. This reaction was reproducible on a larger scale (6 mmol) and gave **3** in 37% yield. Water was insoluble in the organic solvent, but it seemed to cause no problem with the reproducibility. Although we tried to further optimize the conditions, such as amounts of reagents, concentration, temperature and sonication, no better results were obtained. Efforts for optimizations are still being continued.

**Table 1:** The effect of solvents.

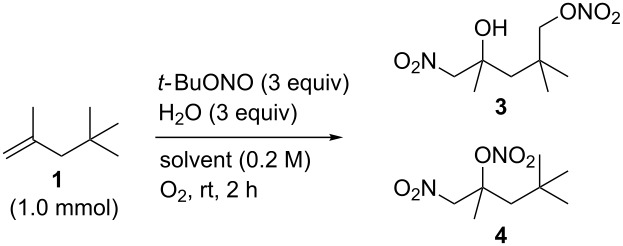			

entry	solvent	yield (%)^a^	
		**3**	**4**

1	DMSO	15	54
2	DMF	15	43
3	THF	18	52
4	CH_2_Cl_2_	13	28
5	pentane	26	28
6^b^	pentane	33 (37)^c^	37 (38)^c^

^a^Isolated yield. ^b^30 equiv of H_2_O, 12 h. ^c^6.0 mmol scale.

Next, our interest focused on the multifunctionalization reactions of various alkenes **5**–**14** (Table 2). The oxynitration of olefins and the direct oxygenation of methyl C–H bonds of branched aliphatic alkenes **5–10** occurred to afford triple functionalized products **15–20** (Table 2, entries 1–6). In the reactions of alkenes **5** and **7**, a remarkable diastereoselectivity was observed (Table 2, entries 1 and 3, and vide infra). We found that the multifunctionalization of monosubstituted alkene **8** took place to give **18** unlike the reaction without water [21], though the yield was not high (entry 4). The reaction of 1,1,3,3-tetramethyl-5-methylenecyclohexane (**9**) gave the mutifunctionalized product **19** as a single isomer. The stereochemistry of **19** could be presumably assigned by considering the 1,5-hydrogen shift mechanism (entry 5) [17]. The reaction of alkene **10** bearing a methyl ester moiety gave γ-lactone **20** by intramolecular transesterification with a tertiary hydroxy group arising from hydration of the olefin (entry 6). Functionalization reactions involving oxygenation of methylene C–H bonds of alkenes **11–13** also proceeded to afford secondary nitrate compounds **21–23**, though no diastereoselectivity was observed (Table 2, entries 7–9). Unfortunately, no tertiary nitrate ester **24** was detected in the reaction of **14**. This implies that the 1,5-hydrogen shift on the methine site is not so fast (entry 10). Typically, in the radical hydrogen abstraction, a methine C–H bond is more reactive than methyl and methylene sites, whereas it might be difficult for substrates having a methine group to form an organized transition state to induce the 1,5-hydrogen shift because they have only one hydrogen atom that can be abstracted [17]. In short, entropy factors might be dominant in the present reaction.

**Table 2:** Reactions of various alkenes^a^.

entry	alkene	product	yield^b^

1	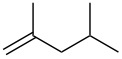 **5**	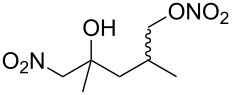 **15**	40%; dr, 90:10
2	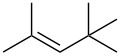 **6**	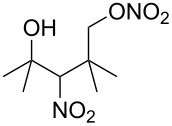 **16**	29%
3	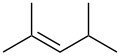 **7**	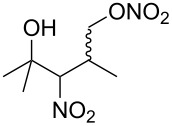 **17**	38%; dr, >95:5
4^c^	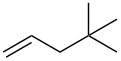 **8**	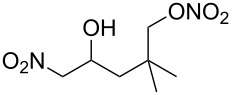 **18**	16%
5^c^	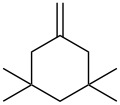 **9**	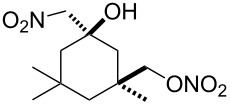 **19**	28%
6^b^	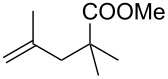 **10**	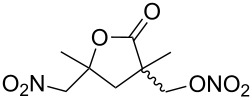 **20**	31%; dr, 50:50
7	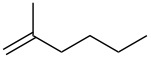 **11**	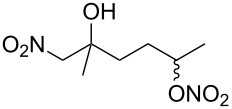 **21**	31%; dr, 50:50
8	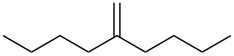 **12**	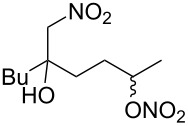 **22**	33%; dr, 50:50
9	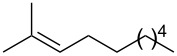 **13**	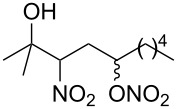 **23**	37%; dr, 50:50
10	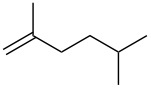 **14**	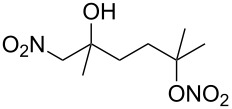 **24**	not detected

^a^Reaction conditions: alkene (1.0 mmol), *t-*BuONO (3.0 mmol), H_2_O (30 mmol) in pentane (5 mL) under an oxygen atmosphere (balloon) for 12 h. ^b^Isolated yield. Diastereomeric ratio (dr) was approximately estimated by ^1^H NMR analysis. ^c^3 equiv (3.0 mmol) of H_2_O was used.

**Scheme 2:**
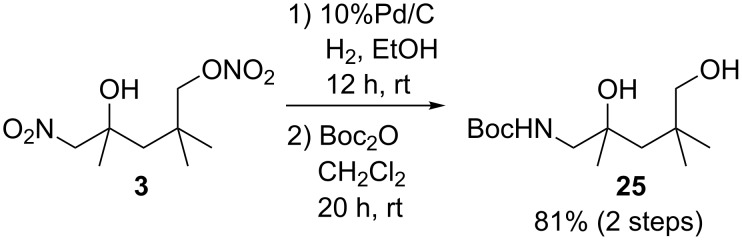
Transformation of **3** into 5-amino-1,4-diol derivative **25**.

We attempted to transform the product obtained by the present reaction into a valuable compound. As a represent example, compound **3** was exposed to typical hydrogenation conditions using 10% palladium on carbon to obtain the corresponding 5-amino-1,4-diol compound. Since this is a highly polar compound, diol **25** was isolated after protection of the amino group by subsequent treatment of the crude product with di-*tert*-butyl dicarbonate (Boc_2_O) (Scheme 2). This experiment emphasised the potential of the present multifunctionalization method because the simple unsaturated hydrocarbon **1** can be converted into the highly polar amino-1,4-diol compound in only two steps.

**Scheme 3:**
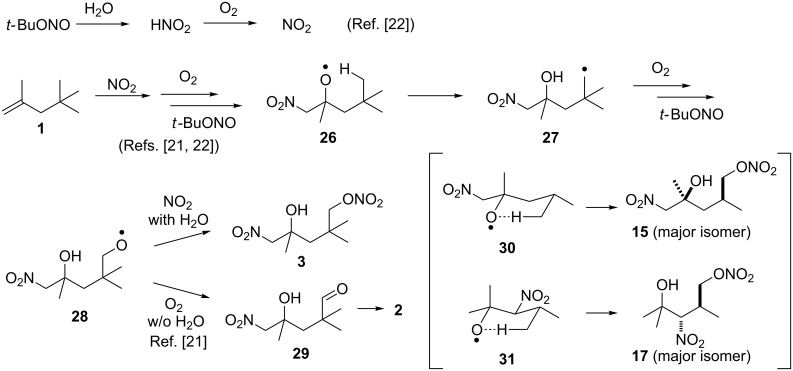
Proposed reaction mechanism.

The plausible mechanism is fundamentally the same as the previously proposed one (Scheme 3) [21–23]. Certainly, the present reaction is triggered by addition of nitrogen dioxide (NO_2_) generated by aerobic decomposition of *t-*BuONO to the olefin of **1**. After the resultant tertiary carbon radical has been trapped by molecular oxygen, an alkoxy radical **26** is formed from the peroxynitrite intermediate (ROONO) generated by the reaction of the peroxy radical (ROO•) intermediate with *t-*BuONO. The alkoxy radical causes an 1,5-hydrogen shift to give the corresponding alkyl radical **27** followed by formation of another alkoxy radical **28** through a similar process. An important issue in the current work is the effect of water to determine the destiny of **28**. In the absence of water, the alkoxy radical **28** was merely oxidized to aldehyde **29**, which was converted into γ-lactol **2** [21]. On the other hand, if **28** is caught by NO_2_ more rapidly than the oxidation, nitrate ester **3** is produced, and it was really observed in the presence of water. These observations clearly indicate that a difference in the concentration of NO_2_ in situ affects the results of each reaction. In other words, water may promote the generation of NO_2_ from *t-*BuONO, and this observation has already been reported in previous radical oxynitration reactions of alkenes [22]. In addition, when alkene **1** was subjected to nitrous acid (HNO_2_), that was formed from sodium nitrite (NaNO_2_; 3 equiv) and acetic acid (3 equiv), and oxygen in water (30 equiv) and pentene (0.2 M), production of compound **3** was observed, though the yield was low (7%). Since this system is an alternative method for the generation of NO_2_, this may support the formation of same intermediate species such as HNO_2_ and NO_2_ in the reaction using *t-*BuONO, oxygen and water [24].

The high diastereoselectivity in reactions of alkenes **5** and **7** could be rationalized by six-membered transition states **30** and **31** in the 1,5-hydrogen shift. If chair forms were postulated in both cases, more substituents should be arranged at equatorial positions to form more stable transition states. According to this presumption, the configurations of the major isomers of products **15** and **17** can be tentatively assigned as shown in Scheme 3.

## Conclusion

We found in multifunctionalization reactions of alkenes with *t-*BuONO and oxygen that water induced the production of another triple functionalized compound that is a nitrate ester. This can be a precursor of a 5-amino-1,4-diol, and the access from simple aliphatic alkenes in one step is unprecedented. The functionalization of unreactive sp^3^ C–H bonds using a radical 1,5-hydrogen shift is an old methodology compared with modern transition metal-catalyzed C–H activation reactions [25–27], but the current work has shown that this old methodology still has a large potential. The development of other direct C–H functionalization reactions based on radical chemistry is currently on-going together with further optimization of the presented reaction in our laboratory.

## Supporting Information

File 1 Experimental details, characterization data of all products, and copies of NMR spectra.

## References

[1] Pellissier H (2013). Chem Rev.

[2] Matsuo Y, Isobe H, Tanaka T, Murata Y, Murata M, Komatsu K, Nakamura E (2005). J Am Chem Soc.

[3] Hilt G, Hess W, Harms K (2006). Org Lett.

[4] Jiang B, Tu S-J, Kaur P, Wever W, Li G (2009). J Am Chem Soc.

[5] Arai S, Koike Y, Hada H, Nishida A (2010). J Am Chem Soc.

[6] Muñiz K, Martínez C (2013). J Org Chem.

[7] Souto J A, Becker P, Iglesias Á, Muñiz K (2012). J Am Chem Soc.

[8] Li Y-M, Sun M, Wang H-L, Tian Q-P, Yang S-D (2013). Angew Chem, Int Ed.

[9] Egami H, Shimizu R, Kawamura S, Sodeoka M (2013). Angew Chem, Int Ed.

[10] Renaud P, Sibi M P (2001). Radicals in Organic Synthesis.

[11] Tojino M, Ryu I, Zhu J, Bienaymé H Free-Radical-Mediated Multicomponent Coupling Reactions. Multicomponent Reactions.

[12] Wille U (2013). Chem Rev.

[13] Recupero F, Punta C (2007). Chem Rev.

[14] Ishihara Y, Baran P S (2010). Synlett.

[15] Chen H, Sanjaya S, Wang Y-F, Chiba S (2013). Org Lett.

[16] Amaoka Y, Nagatomo M, Inoue M (2013). Org Lett.

[17] Čeković Ž (2003). Tetrahedron.

[18] Barton D H R, Beaton J M, Geller L E, Pechet M M (1960). J Am Chem Soc.

[19] Barton D H R, Beaton J M, Geller L E, Pechet M M (1961). J Am Chem Soc.

[20] Wolff M E (1963). Chem Rev.

[21] Taniguchi T, Sugiura Y, Hatta T, Yajima A, Ishibashi H (2013). Chem Commun.

[22] Taniguchi T, Yajima A, Ishibashi H (2011). Adv Synth Catal.

[23] Maity S, Naveen T, Sharma U, Maiti D (2013). Org Lett.

[24] Shiri M, Zolfigol M A, Kruger H G, Tanbakouchian Z (2010). Tetrahedron.

[25] Chen X, Engle K M, Wang D-H, Yu J-Q (2009). Angew Chem, Int Ed.

[26] Lyons T W, Sanford M S (2010). Chem Rev.

[27] Ackermann L (2011). Chem Rev.

