# Supplementary material for: Aerobic radical multifunctionalization of alkenes using tert-butyl nitrite and water
Source: Beilstein J Org Chem. 2013 Aug 20;9:1713–7. doi: 10.3762/bjoc.9.196 (PMC3778400; doi:10.3762/bjoc.9.196)
Supplement: File 1 — Experimental details, characterization data of all products, and copies of NMR spectra. [file Beilstein_J_Org_Chem-09-1713-s001.pdf]

Supporting Information

for

Aerobic radical multifunctionalization of alkenes using *tert*-butyl nitrite and water

Daisuke Hirose[1] and Tsuyoshi Taniguchi*[2]

Address: [1] Graduate School of Natural Science and Technology, Kanazawa University, Kakuma-machi, Kanazawa 920-1192, Japan, and [2]School of Pharmaceutical Sciences, Institute of Medical, Pharmaceutical and Health Sciences, Kanazawa University, Kakuma-machi, Kanazawa 920-1192, Japan

Email: Tsuyoshi Taniguchi* - tsuyoshi@p.kanazawa-u.ac.jp

* Corresponding author

Experimental details, characterization data of all products, and copies of NMR spectra.

Table of Contents

Experimental details S2

References...S7

Copies of NMR spectra of isolated products S8

Experimental details

General methods: All glassware was oven dried. All reagents purchased commercially were used without further purification. All solvents were used after dried by distillation from appropriate dehydrating reagents such as calcium hydride, sodium/benzophenone, or molecular sieves. Deuterated chloroform was passed through an alumina column before the use. ^{1}H and ^{13}C NMR spectra were recorded on JEOL JNM ECA600 (600 MHz and 150 MHz, respectively) spectrometer. Chemical shifts (δ) are quoted relative to tetramethylsilane (^{1}H NMR) and the residual signals of chloroform (^{13}C NMR). Silica gel column chromatography was carried out on silica gel 60N (Kanto Kagaku Co., Ltd., spherical, neutral, 63–210 μm). Mass spectra were recorded on JEOL JMS-700 spectrometers (fast atom bombardment, FAB).

Starting materials: Compounds **1**, **5**, **6**, **7**, **8**, **11** and **14** were commercially available. Compounds **9** [1], **10** [2], **12** [3] and **13** [4] were prepared according to literature procedures.

General procedure for multifunctionalization reactions using *tert*-butyl nitrite, water and oxygen:

To a stirred solution of alkene (1.00 mmol) in pentane (5.0 mL) and were added water (540 mg, 30.0 mmol) and *tert*-butyl nitrite (310 mg, 3.00 mmol), and the mixture was stirred at room temperature under O_2 atmosphere (1 atm, balloon). The mixture was diluted with water and extracted with Et_2O. The organic layer was washed with brine and dried with $MgSO_4$. After the solvent was removed under reduced pressure, the residue was purified by silica gel chromatography (hexane/EtOAc) to give the product. Since several products were unstable, these needed to be analyzed as soon as possible after purification.

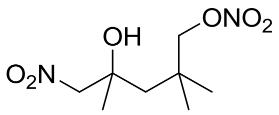

4-Hydroxy-2,2,4-trimethyl-5-nitropentyl nitrate (3) Elute: hexane/EtOAc, 5:1 (second elute). 13–37% yield. colorless oil. ^{1}H NMR (600 MHz, $CDCl_3$) δ 4.51 (d, J = 12.0 Hz, 1H), 4.43 (d, J = 10.2 Hz, 1H), 4.39 (d, J = 10.2 Hz, 1H), 4.38 (d, J = 12.0 Hz, 1H), 3.11 (s, 1H), 1.63 (s, 2H), 1.41 (s, 3H), 1.16 (s, 3H) 1.14 (s, 3H); ^{13}C NMR (150 MHz, $CDCl_3$) δ 85.6, 80.4, 72.3, 46.2, 34.7, 26.8, 26.3; HRFABMS calcd for $C_8H_{17}N_2O_6$ $[M + H]^+$ 237.1087, found: 237.1007.

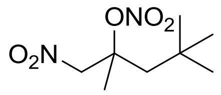

2,4,4-trimethyl-1-nitropent-2-yl nitrate (4) [5] : Elute: hexane/EtOAc, 5:1 (first elute). 28-54% yield. colorless oil. ^{1}H NMR (600 MHz, $CDCl_3$) δ 5.00 (d, *J* = 12.0 Hz, 1H), 4.78 (d, *J* = 12.0 Hz, 1H), 2.13 (d, *J* = 15.0 Hz, 1H), 1.74 (d, *J* = 15.0 Hz, 1H), 1.70 (s, 3H), 1.08 (s, 9H); ^{13}C NMR (150 MHz, $CDCl_3$) δ 88.8, 80.4, 47.0, 31.3, 31.0, 22.0.

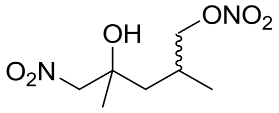

4-Hydroxy-2,4-dimethyl-5-nitropentyl nitrate (15) 12 h. Elute: hexane/EtOAc, 6:1 → 3:1. 86.6 mg, 40% yield (as an inseparable mixture of two diastereomers, ca. 90:10 dr); colorless oil. ^{1}H NMR (600 MHz, $CDCl_3$, for major isomer) δ 4.50 (d, *J* = 12.0 Hz, 1H), 4.43 (d, *J* = 12.0 Hz, 1H), 4.45−4.40 (m, 1H), 4.29 (dd, *J* = 10.8, 6.6 Hz, 1H), 2.99 (s, 1H), 2.27−2.16 (m, 1H), 1.75−1.66 (m, 1H), 1.50−1.43 (m, 1H), 1.37 (s, 3H), 1.12 (d, *J* = 6.9 Hz, 3H); ^{1}H NMR (600 MHz, $CDCl_3$, for partial peaks of minor isomer) δ 4.58 (dd, *J* = 10.8, 5.4 Hz, 1H), 3.04 (s, 1H), 1.35 (s, 3H), 1.10 (d, *J* = 6.9 Hz, 3H); ^{13}C NMR (150 MHz, $CDCl_3$) *δ* 84.8 (minor), 84.4, 77.7, 77.5 (minor), 71.6, 71.5 (minor), 42.2, 42.1 (minor), 27.4, 27.3 (minor), 24.9, 24.1 (minor), 19.0 (minor), 18.6; HRFABMS calcd for $C_7H_{15}N_2O_6$ $[M + H]^+$ 223.0930, found: 223.0923.

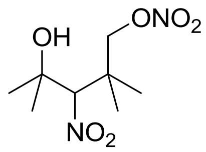

4-Hydroxy-2,2,4-trimethyl-3-nitropentyl nitrate (16) 12 h. Elute: hexane/EtOAc, 5:1. 66.2 mg, 29% yield. colorless oil. ^{1}H NMR (600 MHz, $CDCl_3$) δ 4.73 (d, *J* = 10.8 Hz, 1H), 4.51 (s, 1H), 4.47 (d, *J* = 10.8 Hz, 1H), 2.70 (s, 1H), 1.47 (s, 3H), 1.39 (s, 3H), 1.292−1.288 (m, 6H); ^{13}C NMR (150 MHz, $CDCl_3$) δ 100.0, 77.4, 72.6, 38.5, 30.1, 28.6, 24.9, 23.4; HRFABMS calcd for $C_8H_{17}N_2O_6$ $[M +H]^+$ 237.1087, found: 237.1088.

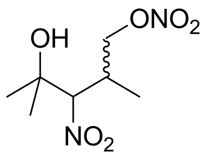

4-Hydroxy-2,4-dimethyl-3-nitropentyl nitrate (17) 12 h. Elute: hexane/EtOAc, 6:1 → 4:1. 82.1 mg, 38% yield (>95:5 dr. Although a trace amount of a minor isomer was detected by ^{1}H NMR analysis of a crude product, it could not be isolated due to the small amount); colorless oil. ^{1}H NMR (600 MHz, $CDCl_3$) δ 4.51−4.49 (m, 3H), 2.77−2.71 (m, 1H), 2.70 (s, 1H), 1.41 (s, 3H), 1.34 (s, 3H), 1.15 (d, *J* = 6.9 Hz, 3H); ^{13}C NMR (150 MHz, $CDCl_3$) δ 94.5, 74.0, 71.5, 31.6, 27.00, 26.98, 13.5; HRFABMS calcd for $C_7H_{15}N_2O_6$ $[M + H]^+$ 223.0930, found: 223.0922.

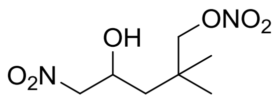

4-Hydroxy-2,2-dimethyl-5-nitro-pentyl nitrate (18) 12 h. Elute: hexane/EtOAc, 5:1 → 3:1. 35.2 mg, 16% yield; colorless oil. ^{1}H NMR (600 MHz, $CDCl_3$) δ 4.48−5.51 (m, 1H), 4.39−4.38 (m, 3H), 4.30 (d, *J* = 10.2 Hz, 1H), 2.74 (br, 1H), 1.66 (dd, *J* = 14.8, 10.3 Hz, 1H), 1.35 (dd, *J* = 14.8, 2.1 Hz, 1H), 1.12 (s, 3H) , 1.10 (s, 3H); ^{13}C NMR (150 MHz, $CDCl_3$) δ 81.1, 80.1, 65.8, 41.4, 33.7, 25.8, 24.2; HRFABMS calcd for $C_7H_{14}N_2O_6$ $[M]^+$ 222.0851, found: 222.0871.

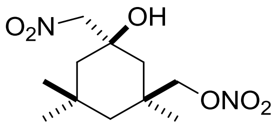

[(1*R,3*S**)-3-Hydroxy-1,5,5-trimethyl-3-nitromethylcyclohexyl]methyl nitrate (19)** 12 h. Elute: hexane/EtOAc, 10:1 → 5:1. 76.2 mg, 28% yield (as a single isomer); colorless oil. ^{1}H NMR (600 MHz, $CDCl_3$) δ 4.77 (d, *J* = 9.6 Hz, 1H), 4.71 (d, *J* = 9.6 Hz, 1H), 4.37 (d, *J* = 12.0 Hz, 1H), 4.33 (d, *J* = 12.0 Hz, 1H), 3.07 (s, 1H), 1.87 (dt, *J* = 14.6, 2.1 Hz, 1H), 1.76 (dt, *J* = 10.7, 2.1 Hz, 1H), 1.64 (dt, *J* = 9.3, 2.4 Hz, 1H), 1.24 (d, *J* = 13.8 Hz, 1H), 1.21 (s, 3H), 1.10 (t, *J* = 13.1 Hz, 2H), 1.03 (s, 3H), 0.97 (s, 3H); ^{13}C NMR (150 MHz, $CDCl_3$) δ 86.3, 78.3, 72.6, 46.8, 45.2, 41.5, 35.9, 34.3, 30.6, 29.9, 28.2; HRFABMS calcd for $C_{11}H_{21}N_2O_6$ $[M + H]^+$ 277.1400, found: 277.1400.

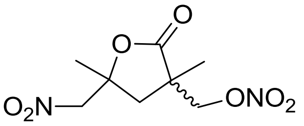

[3,5-Dimethyl-5-(nitromethyl)-2-oxotetrahydrofuran-3-yl]methyl nitrate (20) 12 h. Elute: hexane/EtOAc, 8:1 → 1:1. 75.0 mg, 31% yield (as an inseparable mixture of two diastereomers, ca. 50:50 dr); colorless oil. ^{1}H NMR (600 MHz, $CDCl_3$, for a mixture of

two isomers) δ 4.70−4.56 (m, 3H + 3H), 4.44 (d, J = 10.0 Hz, 1H), 4.38 (d, J = 10.0 Hz, 1H), 2.76 (d, J = 14.4 Hz, 1H), 2.53 (d, J = 14.4 Hz, 1H), 2.36 (d, J = 14.4 Hz, 1H), 2.13 (d, J = 14.1 Hz, 1H), 1.65 (s, 3H), 1.64 (s, 3H), 1.48 (s, 3H), 1.45 (s, 3H); ^{13}C NMR (150 MHz, $CDCl_3$, for a mixture of two isomers) δ 176.4, 176.2, 81.61, 81.53, 79.3, 79.2, 75.8, 75.4, 44.1, 43.8, 40.8, 39.9, 26.6, 26.4, 23.2, 22.3; HRFABMS calcd for $C_8H_{13}N_2O_7$ $[M + H]^+$ 249.0723, found: 249.0723.

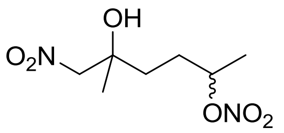

5-Hydroxy-5-methyl-6-nitrohex-2-yl nitrate (21) 12 h. Elute: hexane/EtOAc, 6:1 → 2:1. 62.9 mg, 31% yield (as an inseparable mixture of two diastereomers, ca. 50:50 dr); colorless oil. ^{1}H NMR (600 MHz, $CDCl_3$, for a mixture of two isomers) δ 5.12−5.07 (m, 1H + 1H), 4.47−4.41 (m, 2H + 2H), 2.97 (s, 1H + 1H), 1.86−1.58 (m, 4H + 4H), 1.40−1.38 (m, 3H+3H), 1.328, (s, 3H), 1.320 (s, 3H); ^{13}C NMR (150 MHz, $CDCl_3$, for a mixture of two isomers) δ 84.2, 84.0, 80.9, 80.7, 71.1, 34.7, 34.6, 27.91, 27.85, 24.4, 24.1, 18.5, 18.3; HRFABMS calcd for $C_7H_{15}N_2O_6$ $[M + H]^+$ 223.0930, found: 223.0922.

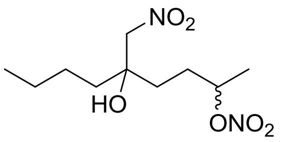

5-Hydroxy-5-nitromethylnon-2-yl nitrate (22) 12 h. Elute: hexane/EtOAc, 5:1 → 4:1. 86.8 mg, 33% yield (as an inseparable mixture of two diastereomers, ca. 50:50 dr); colorless oil. ^{1}H NMR (600 MHz, $CDCl_3$, for a mixture of two isomers) δ 5.09−5.06 (m, 1H + 1H), 4.49−4.42 (m, 2H + 2H), 2.884 (s, 1H), 2.876 (s, 1H), 1.79−1.53 (m, 6H + 6H), 1.39−1.28 (m, 7H + 7H), 0.94−0.91 (m, 3H + 3H); ^{13}C NMR (150 MHz, $CDCl_3$, for a mixture of two isomers) δ 82.5, 82.4, 80.9, 80.8, 73.2, 36.8, 36.5, 32.06, 32.02, 27.7, 27.6, 25.6, 25.5, 22.8, 18.5, 18.3, 13.8; HRFABMS calcd for $C_{10}H_{21}N_2O_6$ $[M + H]^+$ 265.1400, found: 265.1404.

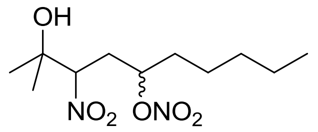

2-Hydroxy-2-methyl-3-nitrodecan-5-yl nitrate (23) 12 h. Elute: hexane/EtOAc, 8:1 → 6:1. 49.2 mg+52.6 mg, 37% yield (as a separable mixture of two diastereomers, ca. 50:50 dr). First elute (**23**): 49.2 mg, 18%; colorless oil. ^{1}H NMR (600 MHz, $CDCl_3$) δ 4.90−4.87 (m, 1H), 4.62−4.59 (m, 1H), 2.59 (ddd, J = 15.8, 11.7, 2.7 Hz, 1H), 2.36 (s,

1H), 2.12 (ddd, J = 16.2, 10.7, 2.7 Hz, 1H), 1.76−1.73 (m, 1H), 1.66−1.63 (m, 1H), 1.37−1.26 (m, 12H), 0.89 (t, J = 7.2 Hz, 3H) ^{13}C NMR (150 MHz, $CDCl_3$) δ 92.3, 80.5, 71.1, 33.1, 31.38, 31.32, 27.2, 26.0, 24.2, 22.3, 13.9; HRFABMS calcd for $C_{11}H_{23}N_2O_6$ $[M + H]^+$ 279.1556, found: 279.1554.

Second elute (**23'**): 52.6 mg, 19%; colorless oil. A small amount of inseparable impurities is detected by ^{1}H and ^{13}C NMR, but the influence on the yield is small because the diasteromeric ratio calculated from the isolated yields is consistent with that estimated by ^{1}H NMR analysis of the crude product. ^{1}H NMR (600 MHz, $CDCl_3$) δ 5.08−5.06 (m, 1H), 4.50 (dd, J = 10.2, 1.8 Hz, 1H), 2.57 (ddd, J = 16.2, 9.6, 6.0 Hz, 1H), 2.35 (s, 1H), 2.28 (ddd, J = 16.2, 6.0, 2.4 Hz, 1H), 1.70−1.64 (m, 2H), 1.40−1.26 (m, 6H), 1.32 (s, 3H), 1.30 (s, 3H), 0.89 (t, J = 6.6 Hz, 3H) ^{13}C NMR (150 MHz, $CDCl_3$) δ 92.6, 81.6, 71.3, 31.9, 31.5, 31.3, 26.6, 26.0, 24.5, 22.3, 13.8; HRFABMS calcd for $C_{11}H_{23}N_2O_6$ $[M + H]^+$ 279.1556, found: 279.1550.

***tert*-Butyl 2,5-dihydroxy-2,4,4-trimethylpentyl carbamate (25):**

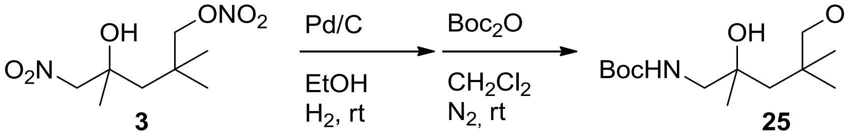

To a solution of **3** (59.0 mg, 0.250 mmol) in EtOH (10.0 mL) was added Pd/C (10%, 20.0 mg) at room temperature, and the mixture was vigorously stirred for 12 h under a hydrogen atmosphere (1 atm, balloon). The reaction mixture was filtered through Celite™ and concentrated under reduced pressure. After the residue was dissolved in CH_2Cl_2 (2.5 mL), Boc_2O (57.2 mg, 0.263 mmol) was added, and the mixture was stirred for 20 h at room temperature. The mixture was diluted with water and extracted with CH_2Cl_2. The organic layer was dried with $MgSO_4$. After the solvent was removed under reduced pressure, the residue was purified by silica gel chromatography (hexane/EtOAc, 2:1) to give the **25** (52.3 mg, 81%) as a colorless oil.

^{1}H NMR (600 MHz, $CDCl_3$) δ 5.11 (br-s, 1H), 4.65 (br, 1H), 4.32 (br, 1H), 3.48 (d, J = 11.0 Hz, 1H), 3.38 (d, J = 11.0 Hz, 1H), 3.19 (dd, J = 14.1, 5.8 Hz, 1H), 3.05 (dd, J = 13.0, 6.9 Hz, 1H), 1.62 (d, J = 15.1 Hz, 1H), 1.45 (s, 9H), 1.38 (d, J = 15.1 Hz, 1H), 1.26 (s, 3H), 1.04 (s, 3H), 0.94 (s, 3H); ^{13}C NMR (150 MHz, $CDCl_3$) δ 157.5, 79.8, 73.7, 71.0, 52.4, 49.3, 36.3, 29.0, 28.3, 26.4, 25.7; HRFABMS calcd for $C_{13}H_{28}NO_4$ $[M + H]^+$ 262.2018, found: 262.2011.

References

1) Alexander, C. W.; Jackson, W. R.; Jennings, W. B. *J. Chem. Soc. B* **1971**, 2241−2243.

2) Formica, C.; Musco, A.; Pontellini, R. *J. Mol. Catal.* **1993**, *84*, 239−251.

3) Wu, F. -L.; Ross, B. P.; McGeary, R. P. *Eur. J. Org. Chem*. **2010**, 1989−1998.

4) Riveiros, R.; Saya, L.; Sestelo, J. P.; Sarandeses, L. A. *Eur. J. Org. Chem*. **2008**, 1959−1966.

5) Taniguchi, T.; Sugiura, Y.; Hatta, T.; Yajima, A.; Ishibashi, H. *Chem. Commun.* **2013**, *49*, 2198−2200.

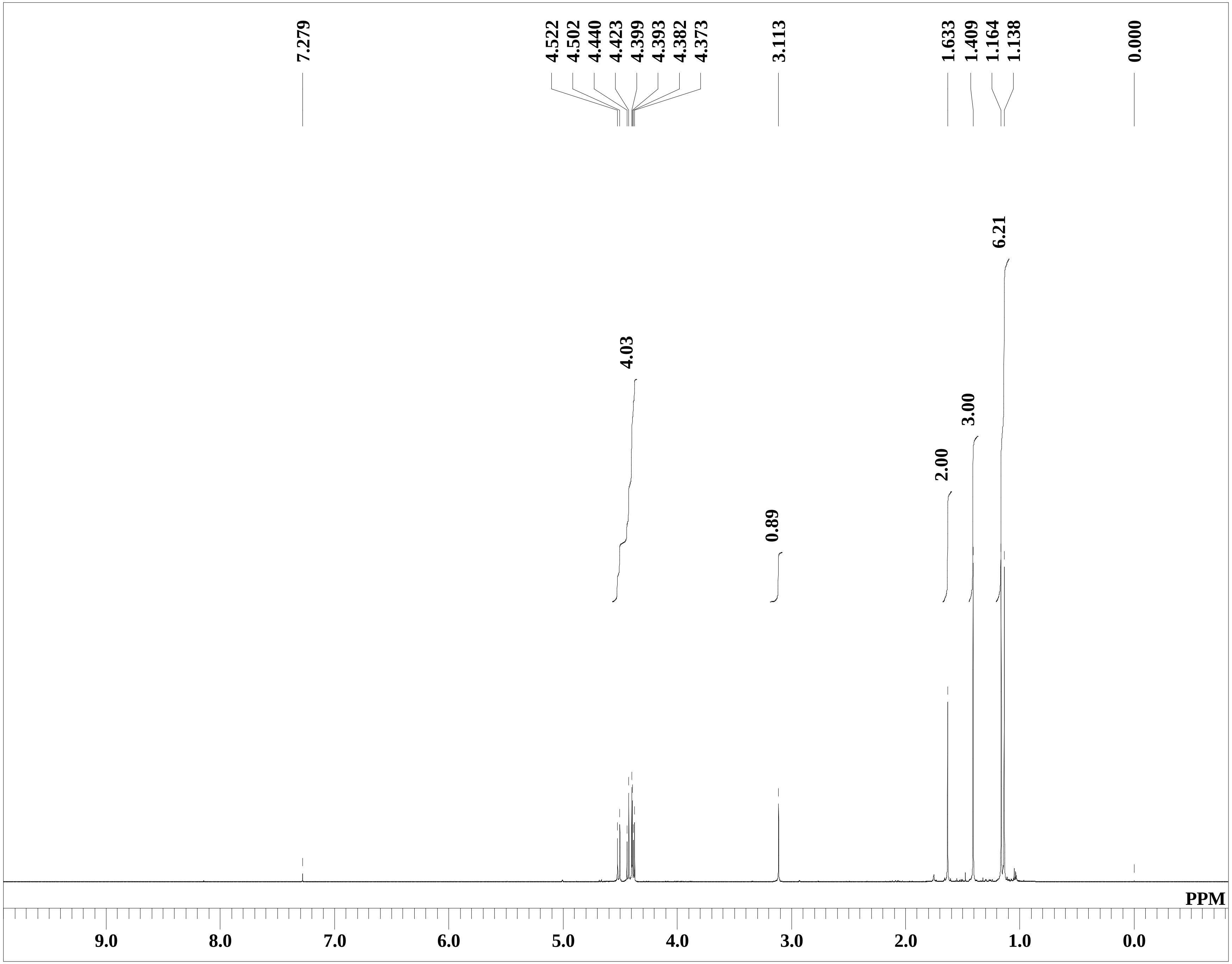
7.279
4.522
4.502
4.440
4.423
4.399
4.393
4.382
4.373
3.113
1.633
1.409
1.164
1.138
0.000
4.03
0.89
2.00
3.00
6.21
PPM
9.0
8.0
7.0
6.0
5.0
4.0
3.0
2.0
1.0
0.0

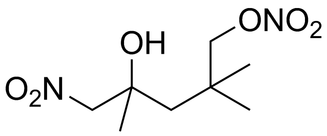
OH
ONO2
O2N

3

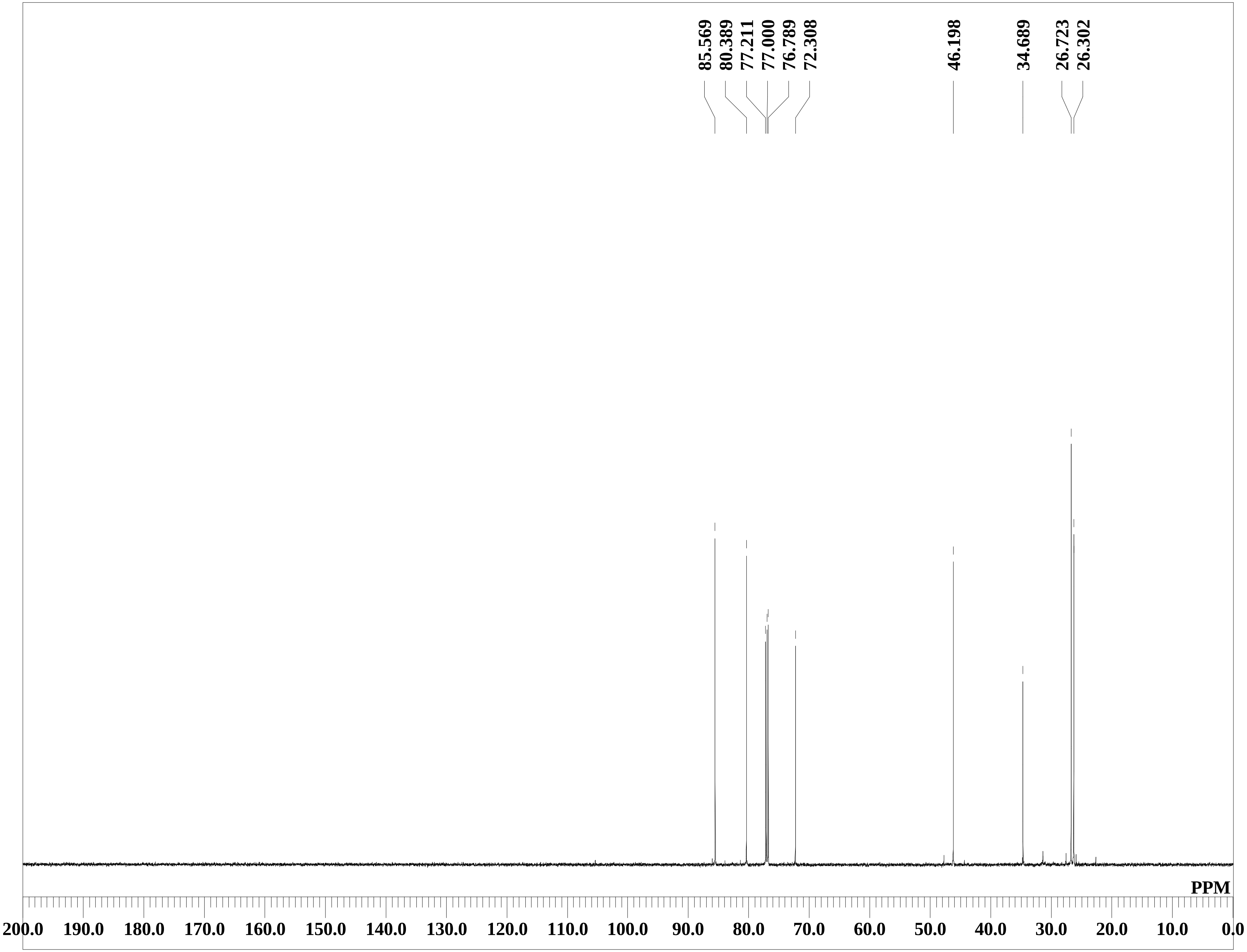
85.569
80.389
77.211
77.000
76.789
72.308
46.198
34.689
26.723
26.302
PPM
200.0 190.0 180.0 170.0 160.0 150.0 140.0 130.0 120.0 110.0 100.0 90.0 80.0 70.0 60.0 50.0 40.0 30.0 20.0 10.0 0.0

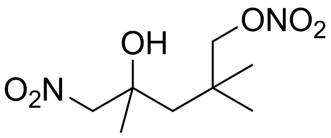
OH
ONO2
O2N

3

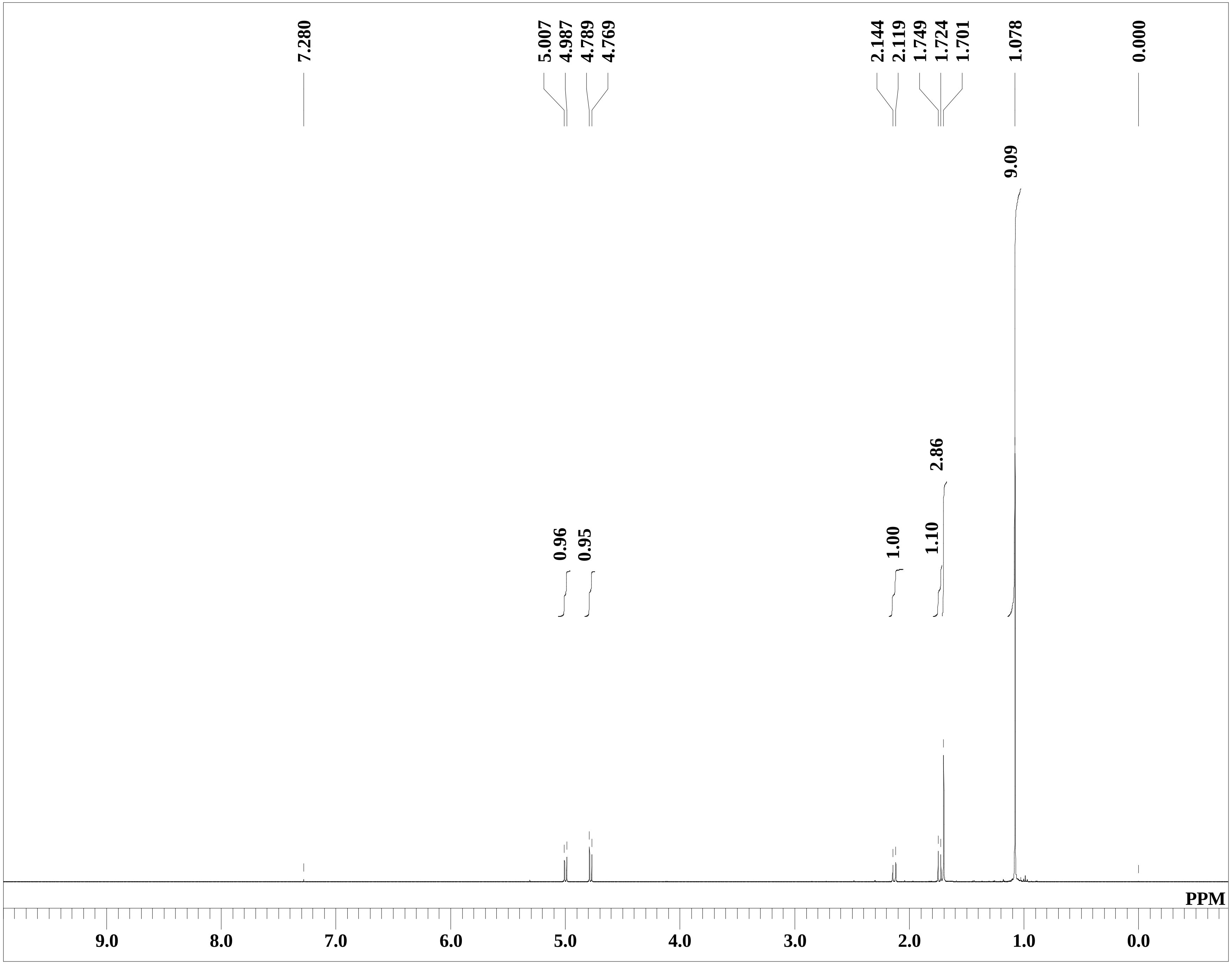
7.280
5.007
4.987
4.789
4.769
2.144
2.119
1.749
1.724
1.701
1.078
0.000
9.09
2.86
0.96
0.95
1.00
1.10
PPM
9.0
8.0
7.0
6.0
5.0
4.0
3.0
2.0
1.0
0.0

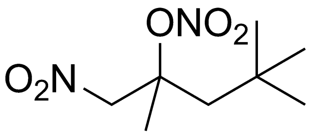
ONO2
O2N

4

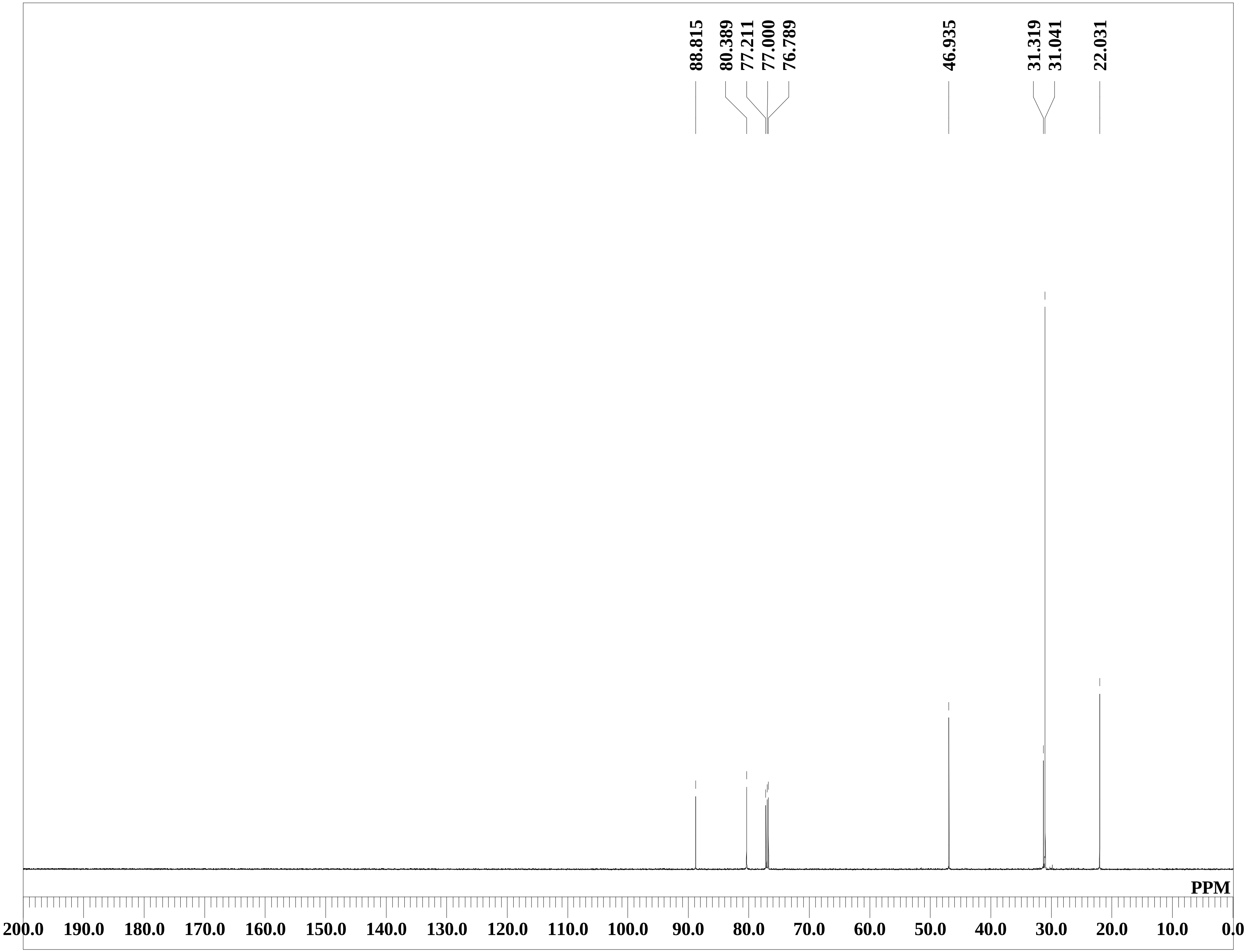

88.815
80.389
77.211
77.000
76.789
46.935
31.319
31.041
22.031
PPM
200.0
190.0
180.0
170.0
160.0
150.0
140.0
130.0
120.0
110.0
100.0
90.0
80.0
70.0
60.0
50.0
40.0
30.0
20.0
10.0
0.0

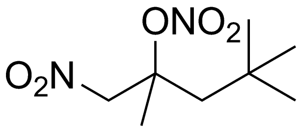

ONO2
O2N

4

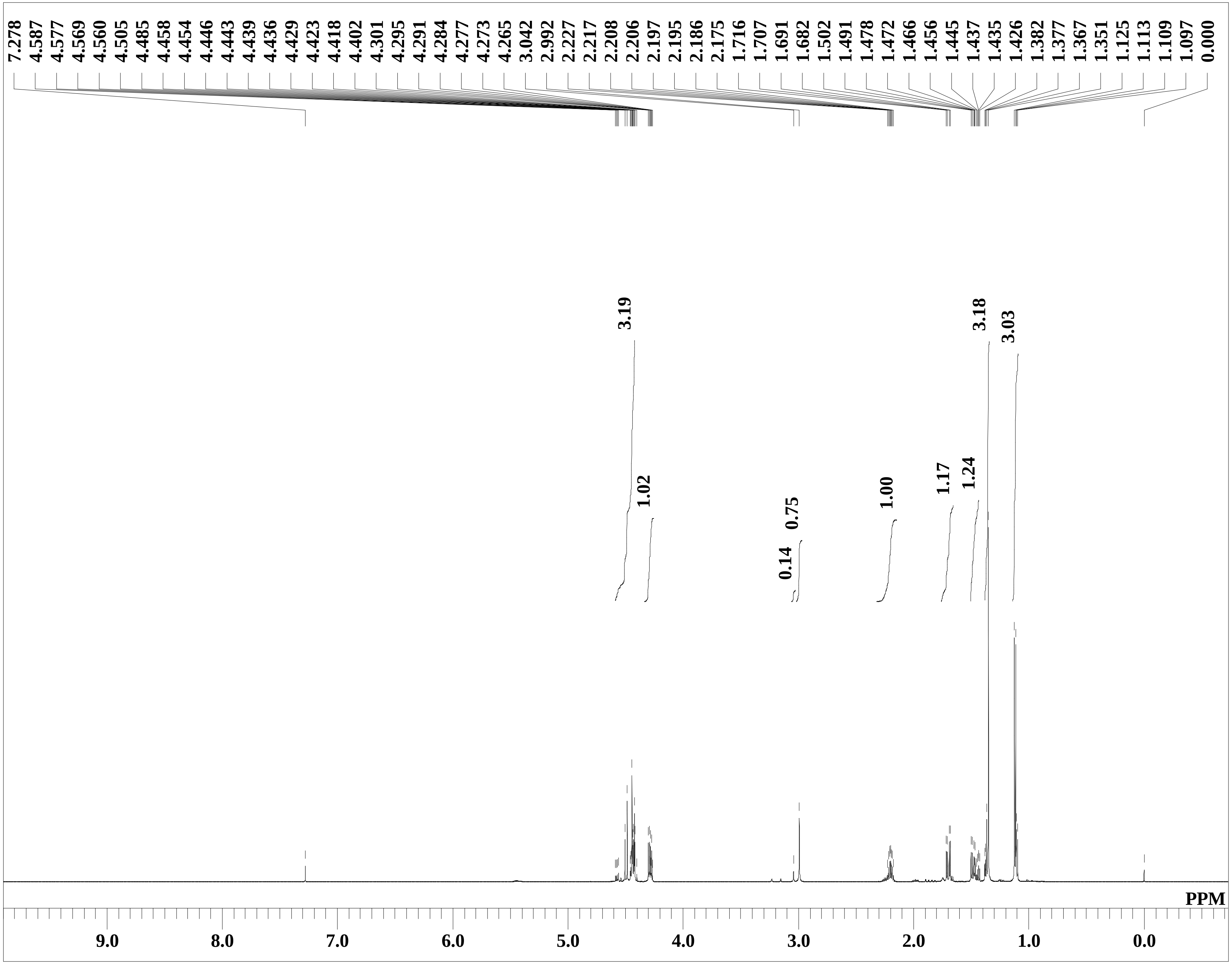
7.278
4.587
4.577
4.569
4.560
4.505
4.485
4.458
4.454
4.446
4.443
4.439
4.436
4.429
4.423
4.418
4.402
4.301
4.295
4.291
4.284
4.277
4.273
4.265
3.042
2.992
2.227
2.217
2.208
2.206
2.197
2.195
2.186
2.175
1.716
1.707
1.691
1.682
1.502
1.491
1.478
1.472
1.466
1.456
1.445
1.437
1.435
1.426
1.382
1.377
1.367
1.351
1.125
1.113
1.109
1.097
0.000
3.19
1.02
0.14
0.75
1.00
1.17
1.24
3.18
3.03
PPM
9.0
8.0
7.0
6.0
5.0
4.0
3.0
2.0
1.0
0.0

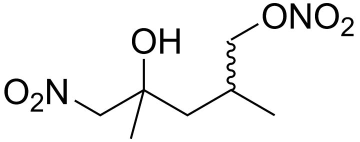
OH
ONO2
O2N

15

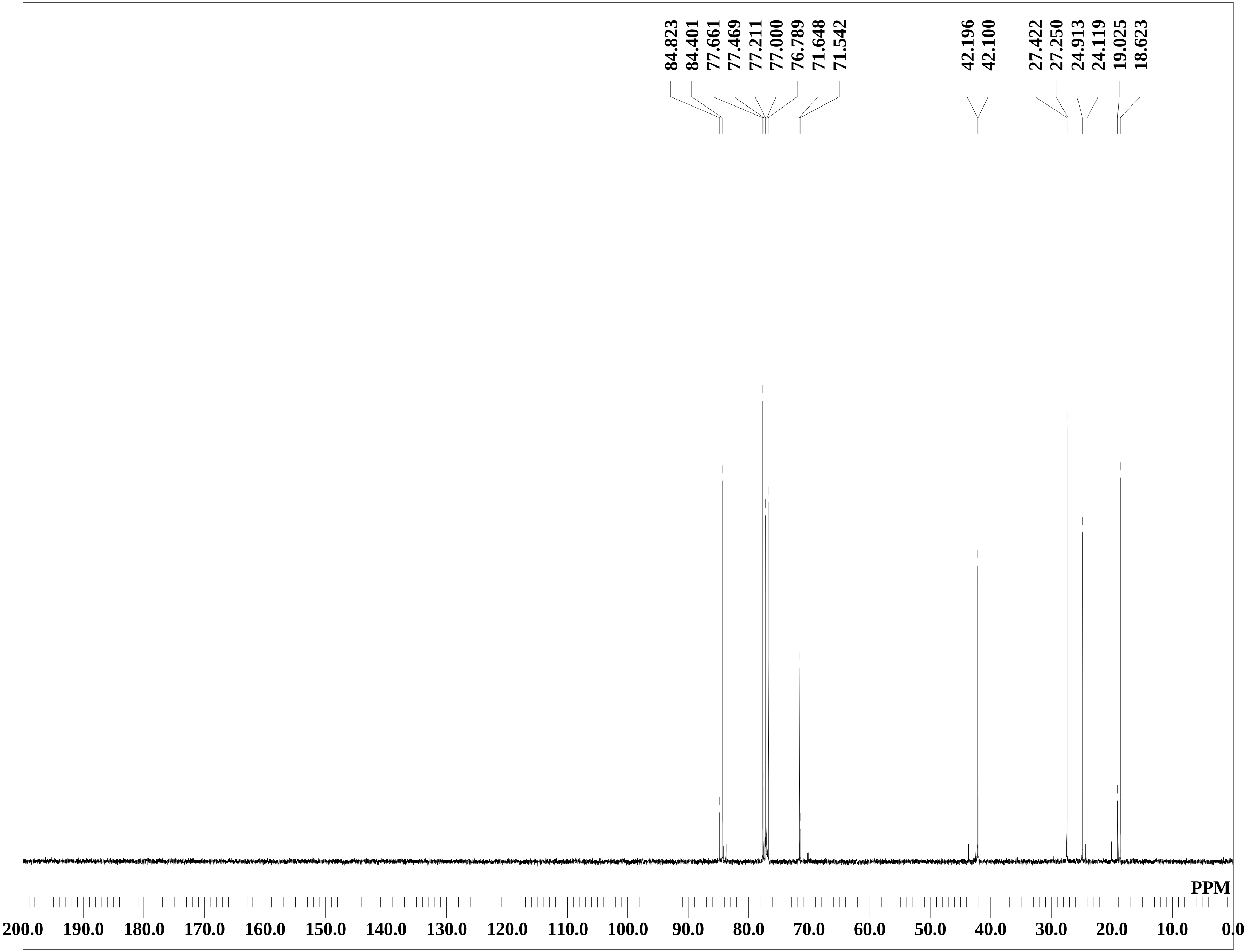
84.823
84.401
77.661
77.469
77.211
77.000
76.789
71.648
71.542
42.196
42.100
27.422
27.250
24.913
24.119
19.025
18.623
PPM
200.0 190.0 180.0 170.0 160.0 150.0 140.0 130.0 120.0 110.0 100.0 90.0 80.0 70.0 60.0 50.0 40.0 30.0 20.0 10.0 0.0

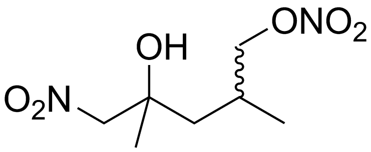
OH
ONO2
O2N

15

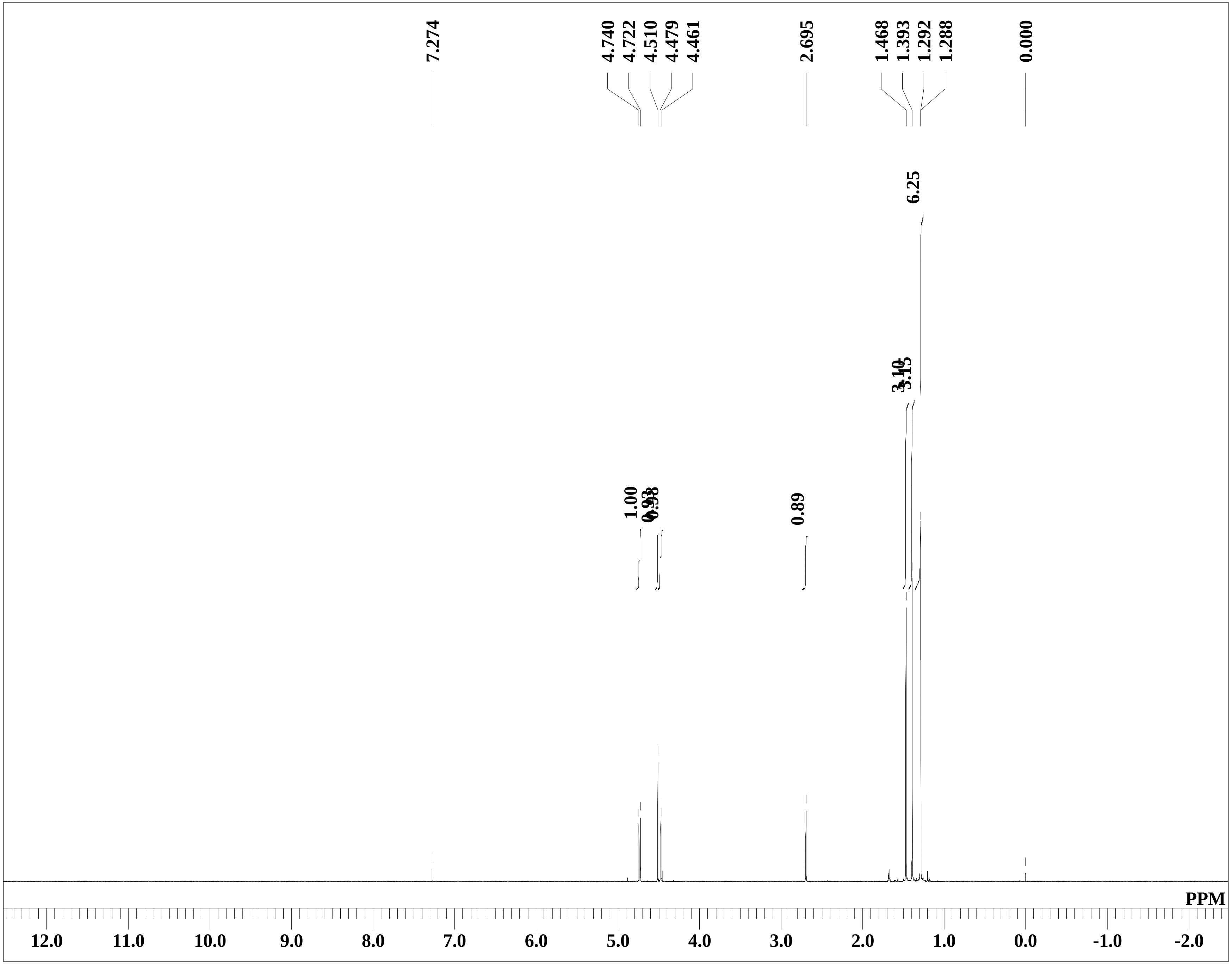
7.274
4.740
4.722
4.510
4.479
4.461
2.695
1.468
1.393
1.292
1.288
0.000
6.25
3.10
3.15
1.00
0.93
0.98
0.89
PPM
12.0
11.0
10.0
9.0
8.0
7.0
6.0
5.0
4.0
3.0
2.0
1.0
0.0
-1.0
-2.0

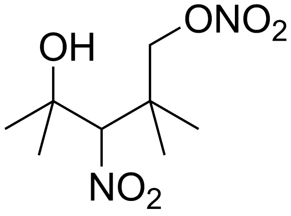
OH
ONO2
NO2

16

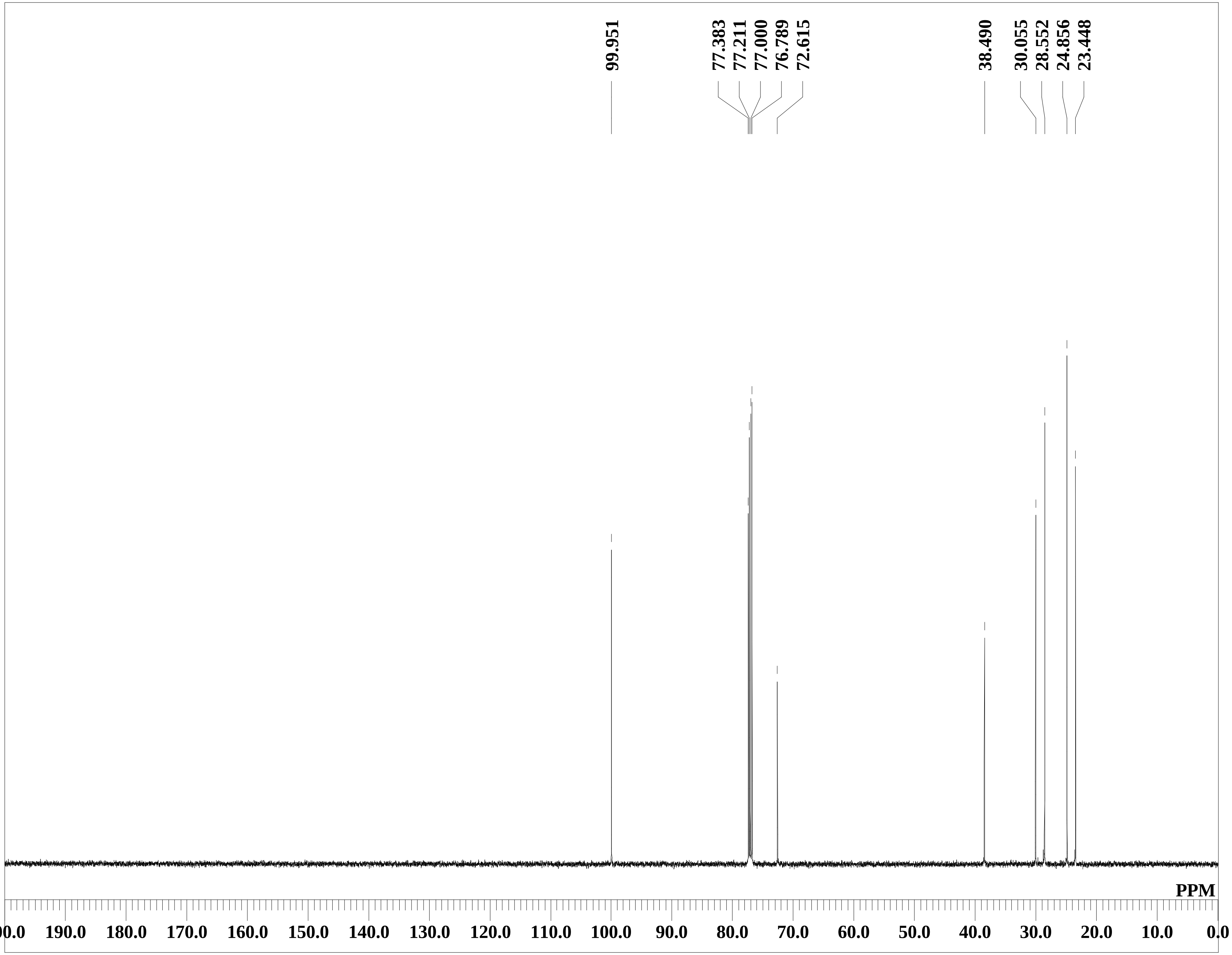
99.951
77.383
77.211
77.000
76.789
72.615
38.490
30.055
28.552
24.856
23.448
PPM
200.0 190.0 180.0 170.0 160.0 150.0 140.0 130.0 120.0 110.0 100.0 90.0 80.0 70.0 60.0 50.0 40.0 30.0 20.0 10.0 0.0

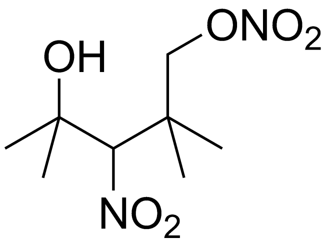
OH
ONO2
NO2

16

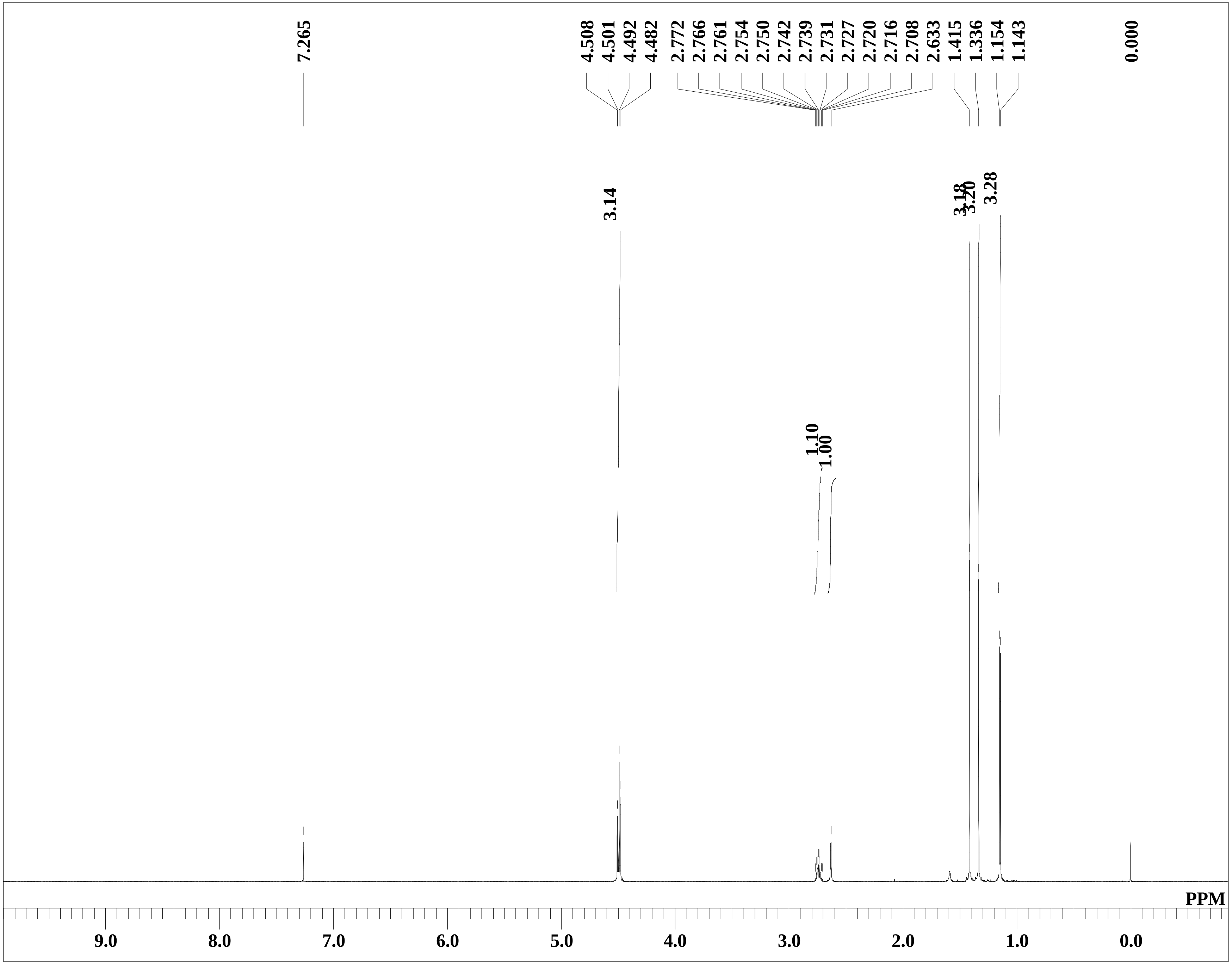
7.265
4.508
4.501
4.492
4.482
2.772
2.766
2.761
2.754
2.750
2.742
2.739
2.731
2.727
2.720
2.716
2.708
2.633
1.415
1.336
1.154
1.143
0.000
3.14
1.10
1.00
3.18
3.20
3.28
PPM
9.0
8.0
7.0
6.0
5.0
4.0
3.0
2.0
1.0
0.0

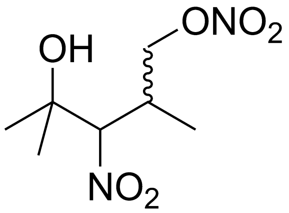
OH
ONO2
NO2

17

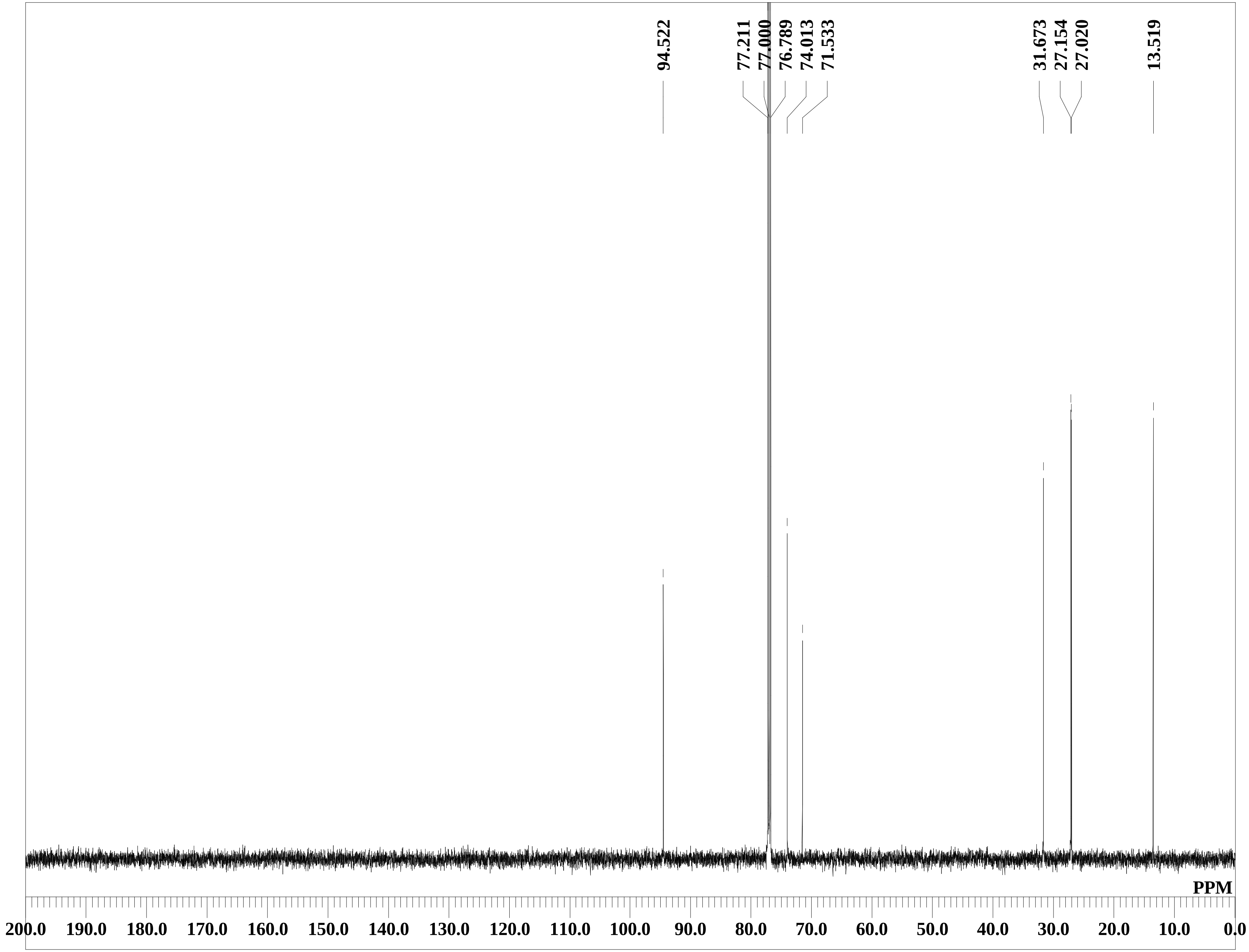
94.522
77.211
77.000
76.789
74.013
71.533
31.673
27.154
27.020
13.519
PPM
200.0
190.0
180.0
170.0
160.0
150.0
140.0
130.0
120.0
110.0
100.0
90.0
80.0
70.0
60.0
50.0
40.0
30.0
20.0
10.0
0.0

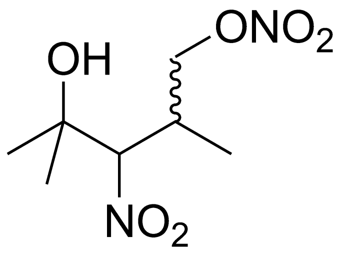
OH
ONO2
NO2

17

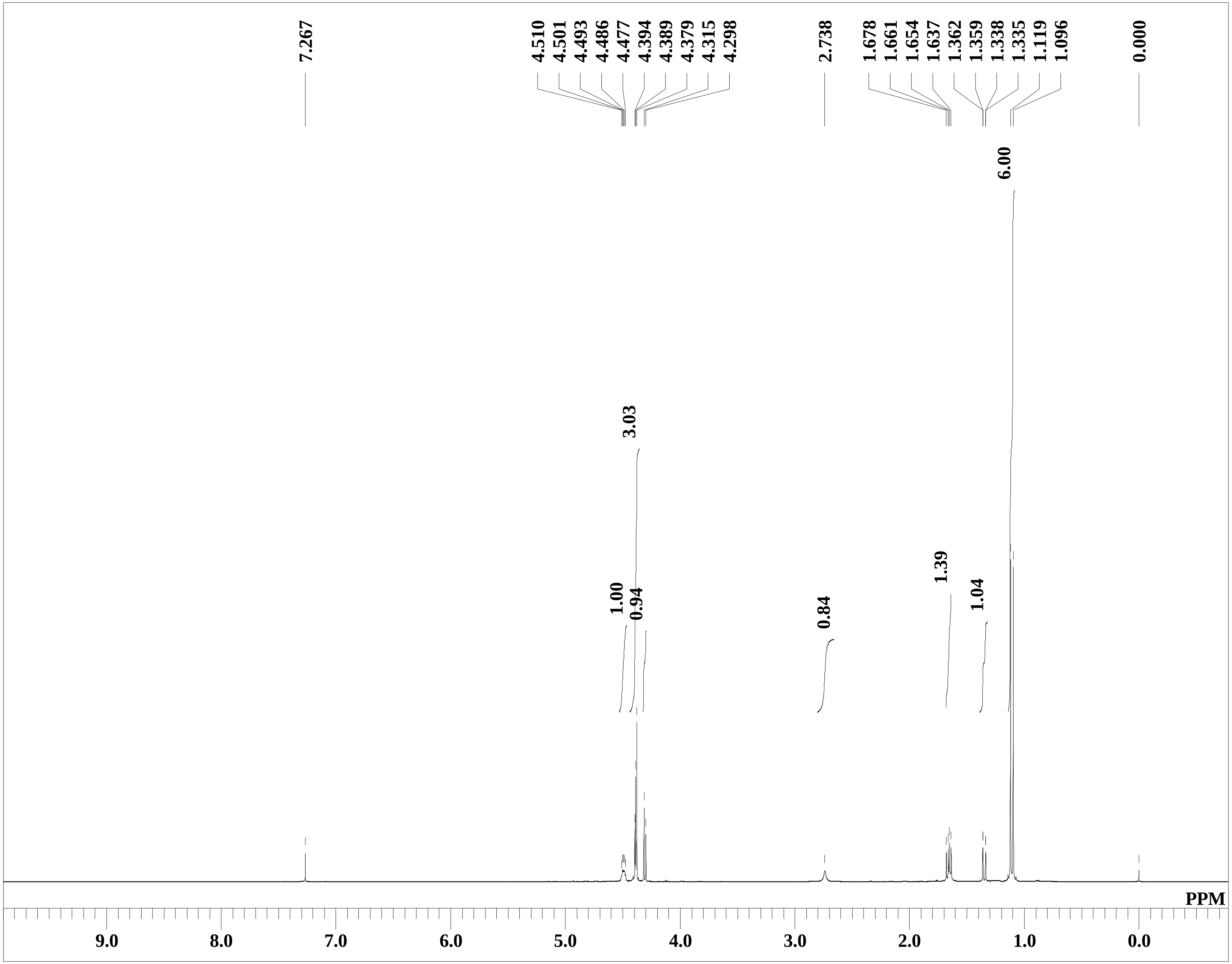

7.267
4.510
4.501
4.493
4.486
4.477
4.394
4.389
4.379
4.315
4.298
2.738
1.678
1.661
1.654
1.637
1.362
1.359
1.338
1.335
1.119
1.096
0.000
6.00
3.03
1.00
0.94
0.84
1.39
1.04
PPM
9.0
8.0
7.0
6.0
5.0
4.0
3.0
2.0
1.0
0.0

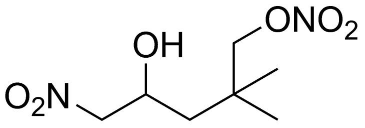

OH
ONO2
O2N

18

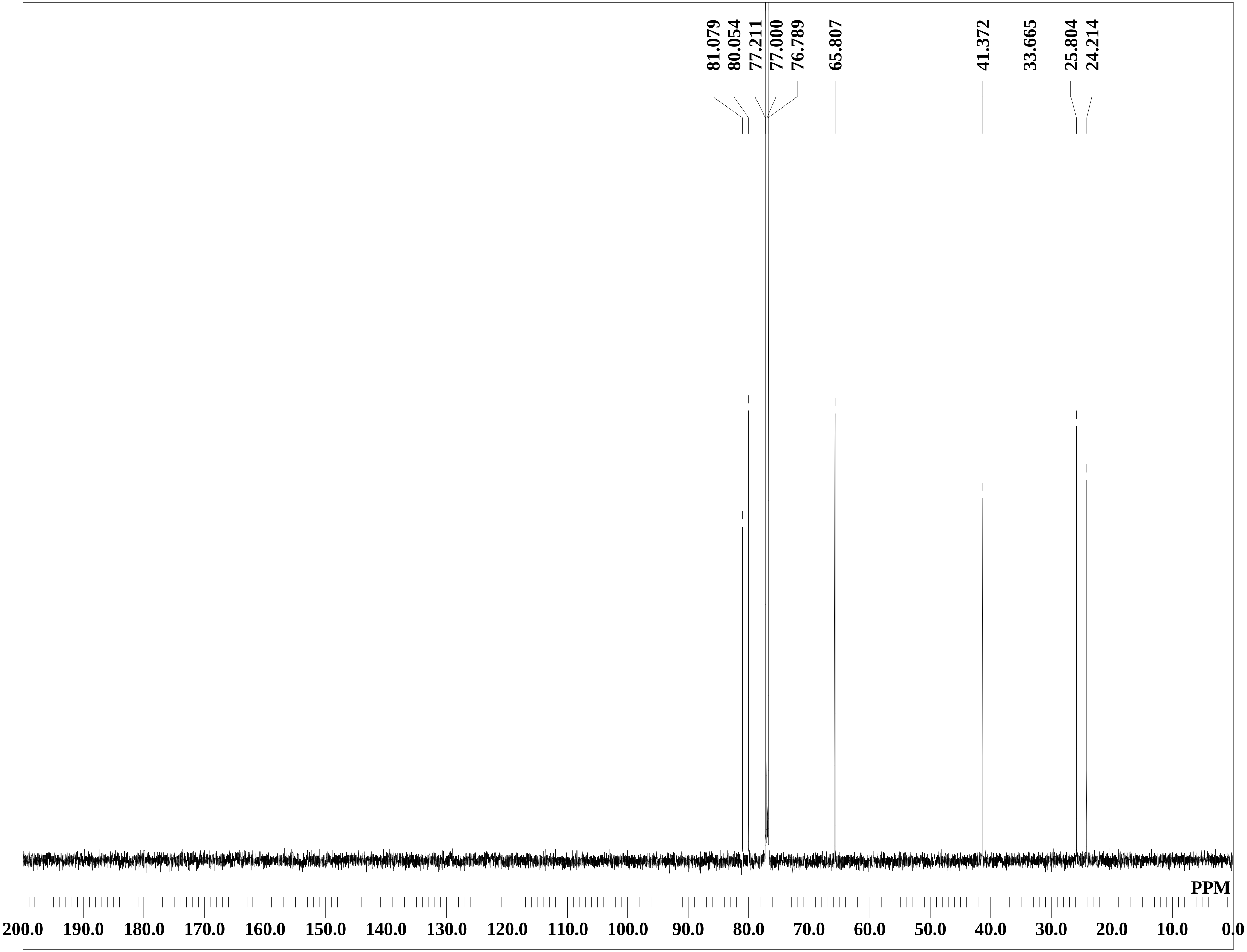
81.079
80.054
77.211
77.000
76.789
65.807
41.372
33.665
25.804
24.214
PPM
200.0 190.0 180.0 170.0 160.0 150.0 140.0 130.0 120.0 110.0 100.0 90.0 80.0 70.0 60.0 50.0 40.0 30.0 20.0 10.0 0.0

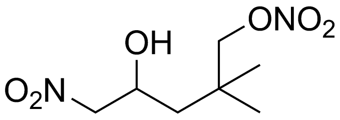
OH
ONO2
O2N

18

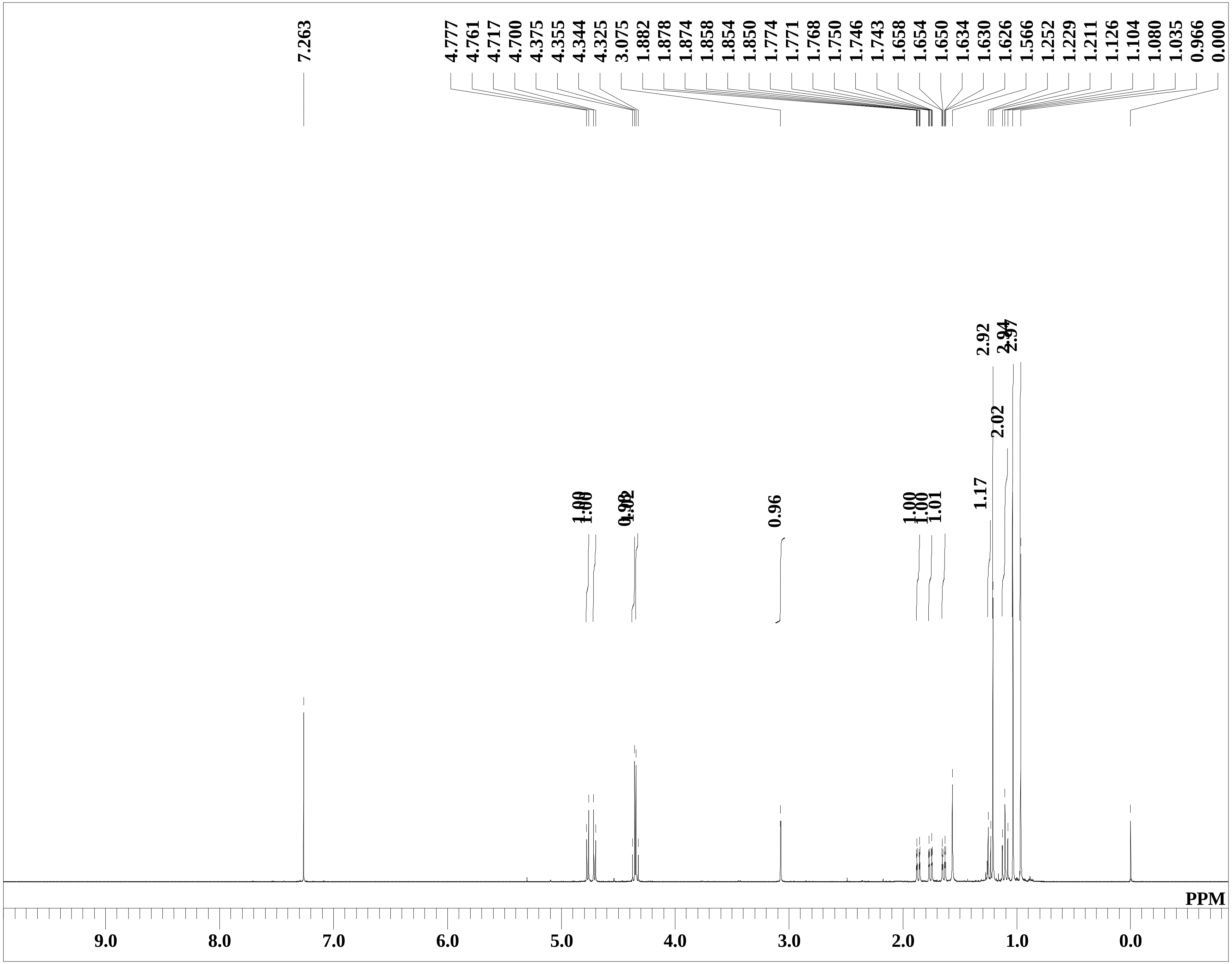
7.263
4.777
4.761
4.717
4.700
4.375
4.355
4.344
4.325
3.075
1.882
1.878
1.874
1.858
1.854
1.850
1.774
1.771
1.768
1.750
1.746
1.743
1.658
1.654
1.650
1.634
1.630
1.626
1.566
1.252
1.229
1.211
1.126
1.104
1.080
1.035
0.966
0.000
1.00
1.00
0.98
1.02
0.96
1.00
1.00
1.01
1.17
2.92
2.02
2.94
2.97
PPM
9.0
8.0
7.0
6.0
5.0
4.0
3.0
2.0
1.0
0.0

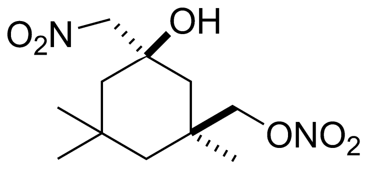
O2N
OH
ONO2

19

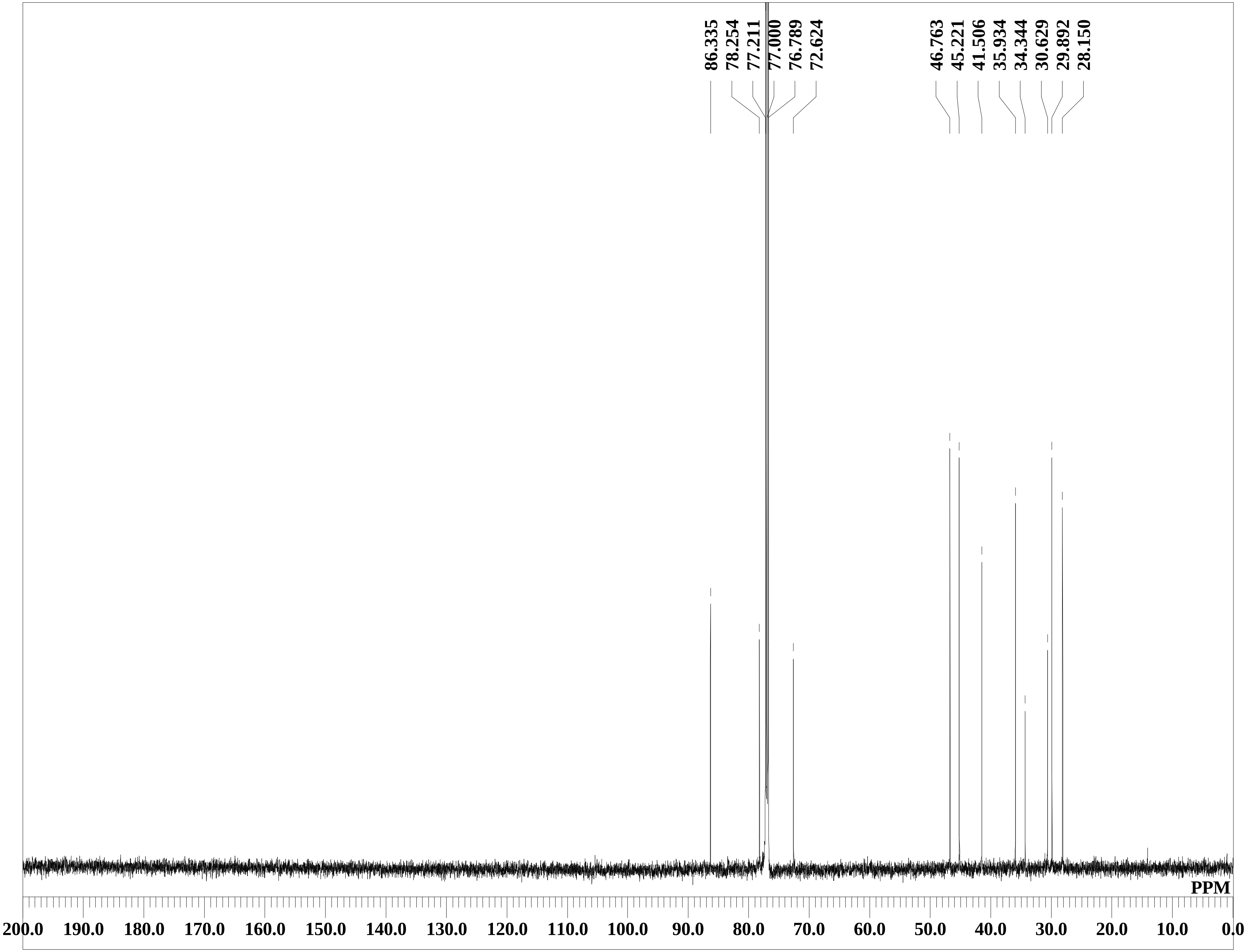
86.335
78.254
77.211
77.000
76.789
72.624
46.763
45.221
41.506
35.934
34.344
30.629
29.892
28.150
PPM
200.0 190.0 180.0 170.0 160.0 150.0 140.0 130.0 120.0 110.0 100.0 90.0 80.0 70.0 60.0 50.0 40.0 30.0 20.0 10.0 0.0

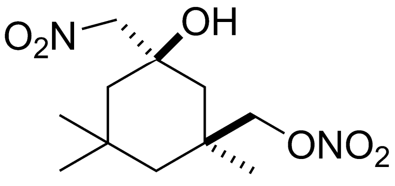
O2N
OH
ONO2

19

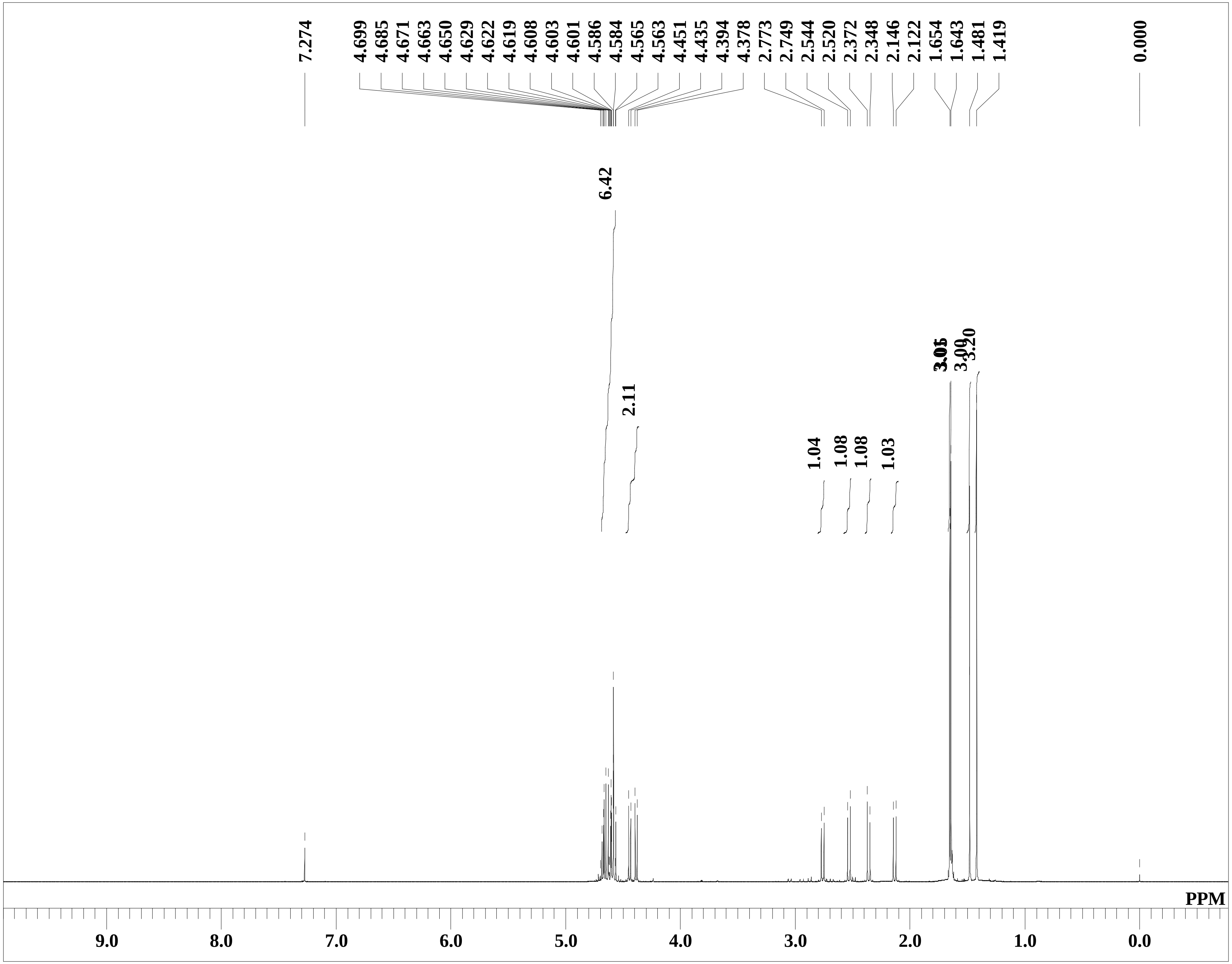
7.274
4.699
4.685
4.671
4.663
4.650
4.629
4.622
4.619
4.608
4.603
4.601
4.586
4.584
4.565
4.563
4.451
4.435
4.394
4.378
2.773
2.749
2.544
2.520
2.372
2.348
2.146
2.122
1.654
1.643
1.481
1.419
0.000
6.42
2.11
1.04
1.08
1.08
1.03
3.05
3.01
3.00
3.20
PPM
9.0
8.0
7.0
6.0
5.0
4.0
3.0
2.0
1.0
0.0

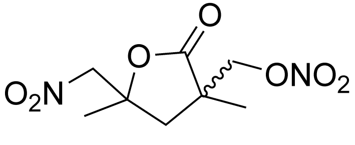
O
O
O_2N
ONO_2

20

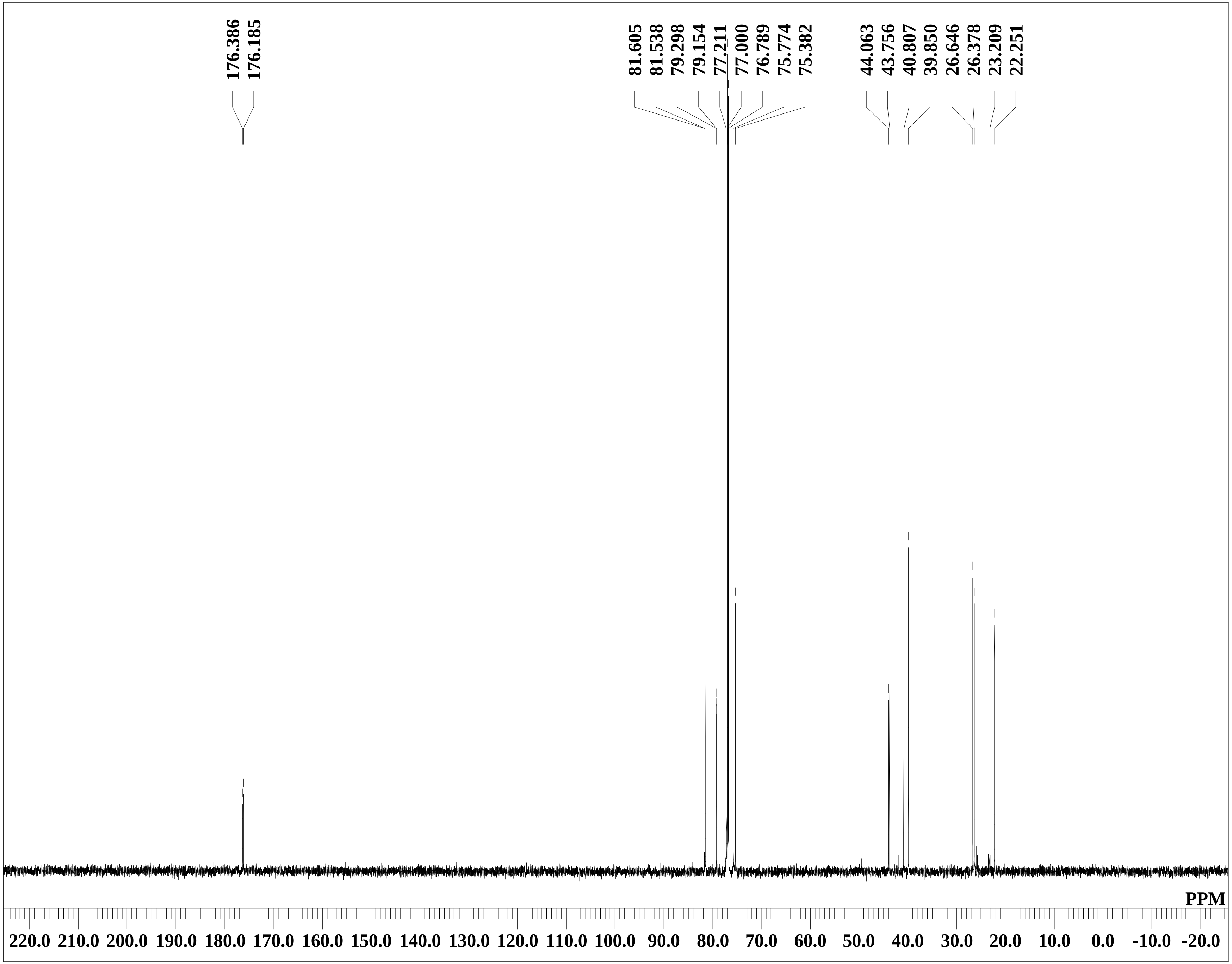
176.386
176.185
81.605
81.538
79.298
79.154
77.211
77.000
76.789
75.774
75.382
44.063
43.756
40.807
39.850
26.646
26.378
23.209
22.251
PPM
220.0 210.0 200.0 190.0 180.0 170.0 160.0 150.0 140.0 130.0 120.0 110.0 100.0 90.0 80.0 70.0 60.0 50.0 40.0 30.0 20.0 10.0 0.0 -10.0 -20.0

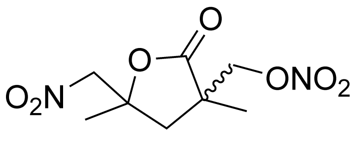
O
O
O_2N
ONO_2

20

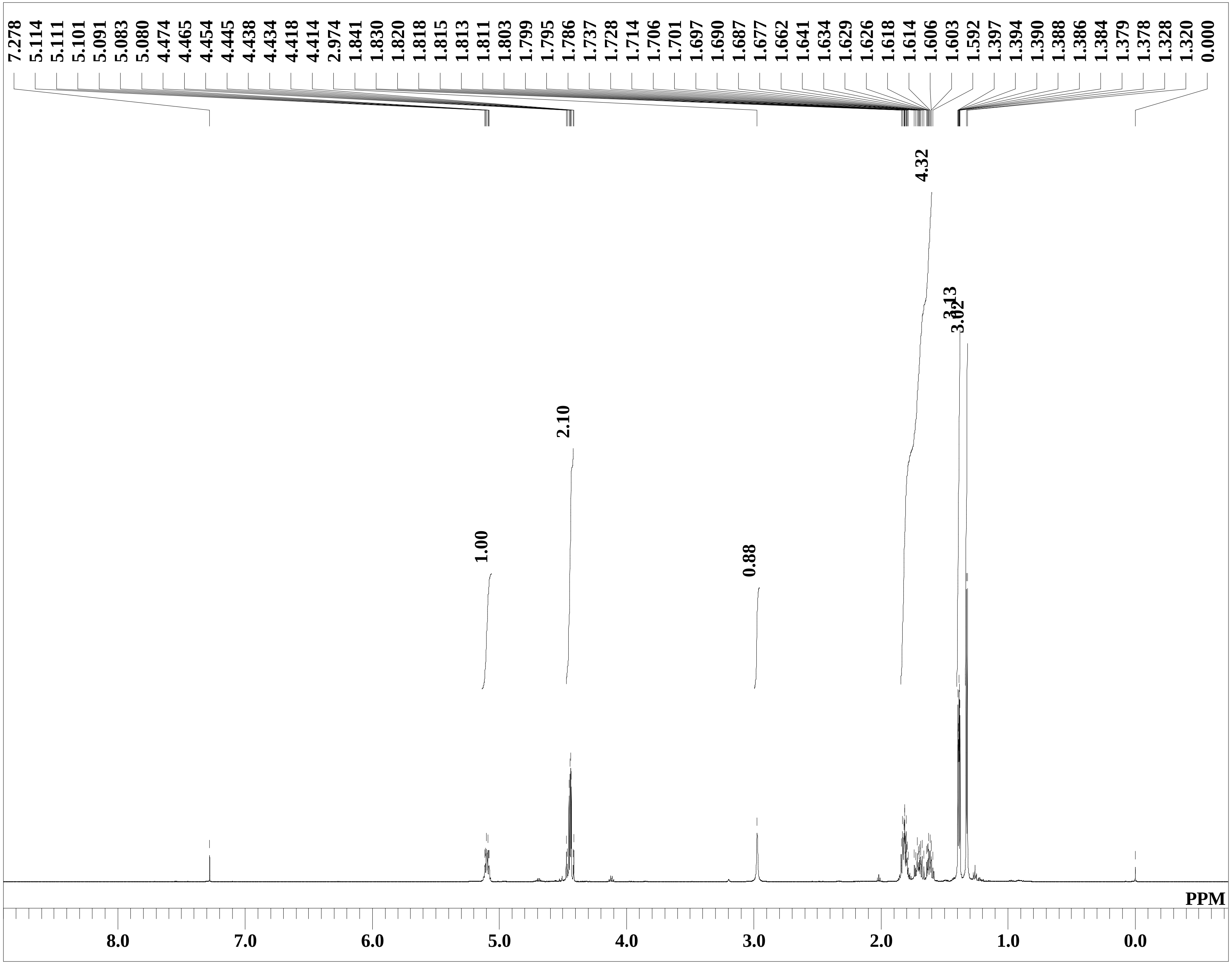
7.278 5.114 5.111 5.101 5.091 5.083 5.080 4.474 4.465 4.454 4.445 4.438 4.434 4.418 4.414 2.974 1.841 1.830 1.820 1.818 1.815 1.813 1.811 1.803 1.799 1.795 1.786 1.737 1.728 1.714 1.706 1.701 1.697 1.690 1.687 1.677 1.662 1.641 1.634 1.629 1.626 1.618 1.614 1.606 1.603 1.592 1.397 1.394 1.390 1.388 1.386 1.384 1.379 1.378 1.328 1.320 0.000
4.32
3.13
3.02
2.10
1.00
0.88
PPM
8.0
7.0
6.0
5.0
4.0
3.0
2.0
1.0
0.0

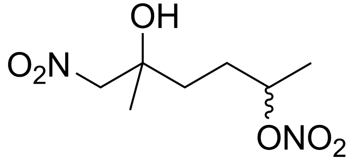
OH
O_2N
ONO_2

21

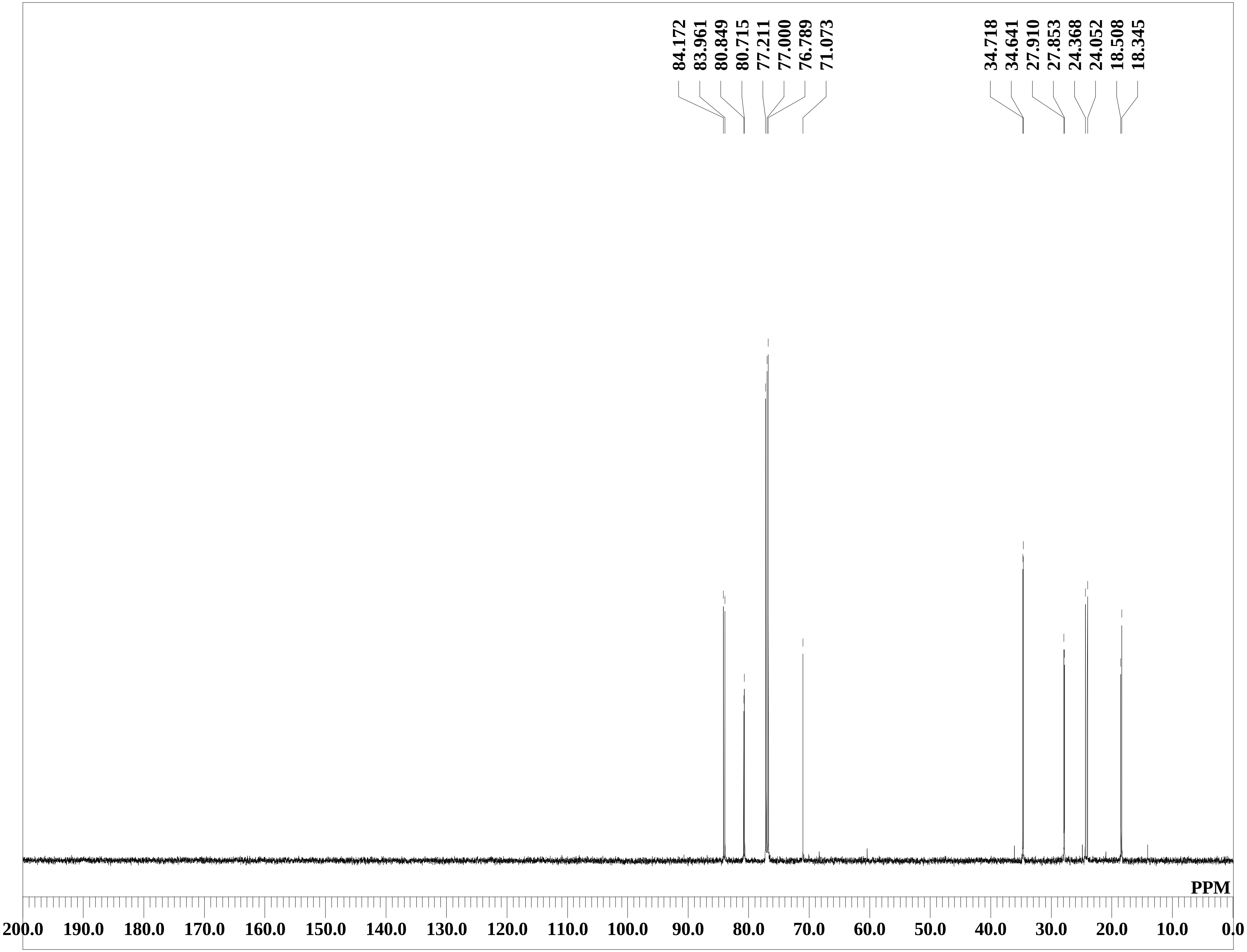
84.172
83.961
80.849
80.715
77.211
77.000
76.789
71.073
34.718
34.641
27.910
27.853
24.368
24.052
18.508
18.345
PPM
200.0
190.0
180.0
170.0
160.0
150.0
140.0
130.0
120.0
110.0
100.0
90.0
80.0
70.0
60.0
50.0
40.0
30.0
20.0
10.0
0.0

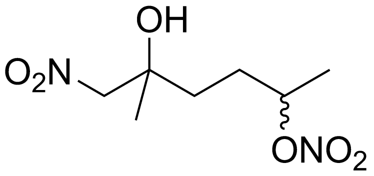
OH
O2N
ONO2

21

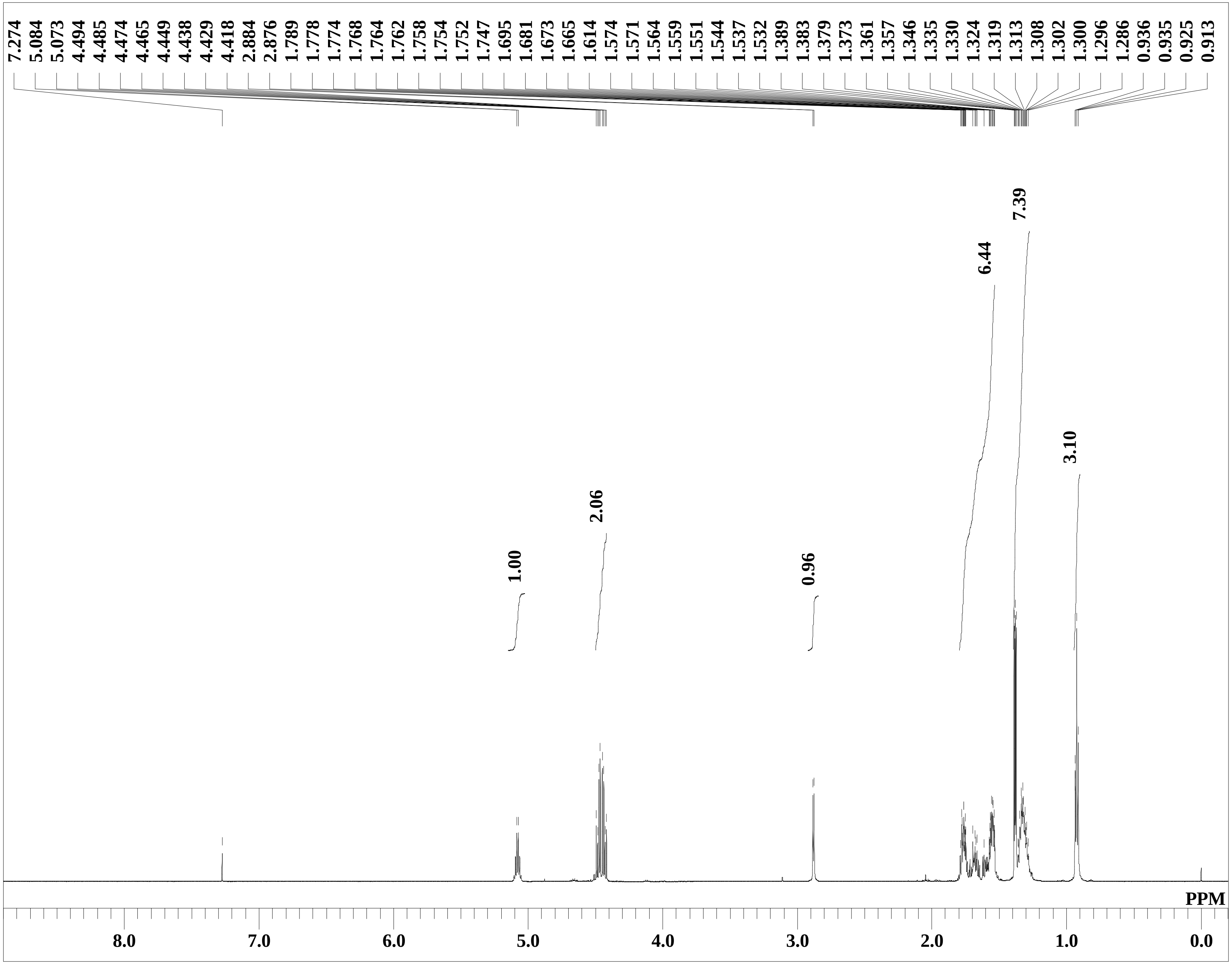
7.274
5.084
5.073
4.494
4.485
4.474
4.465
4.449
4.438
4.429
4.418
2.884
2.876
1.789
1.778
1.774
1.768
1.764
1.762
1.758
1.754
1.752
1.747
1.695
1.681
1.673
1.665
1.614
1.574
1.571
1.564
1.559
1.551
1.544
1.537
1.532
1.389
1.383
1.379
1.373
1.361
1.357
1.346
1.335
1.330
1.324
1.319
1.313
1.308
1.302
1.300
1.296
1.286
0.936
0.935
0.925
0.913
1.00
2.06
0.96
6.44
7.39
3.10
PPM
8.0
7.0
6.0
5.0
4.0
3.0
2.0
1.0
0.0

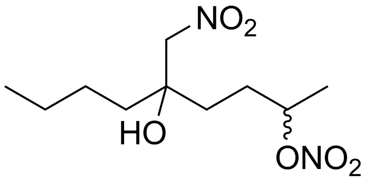
NO2
HO
ONO2

22

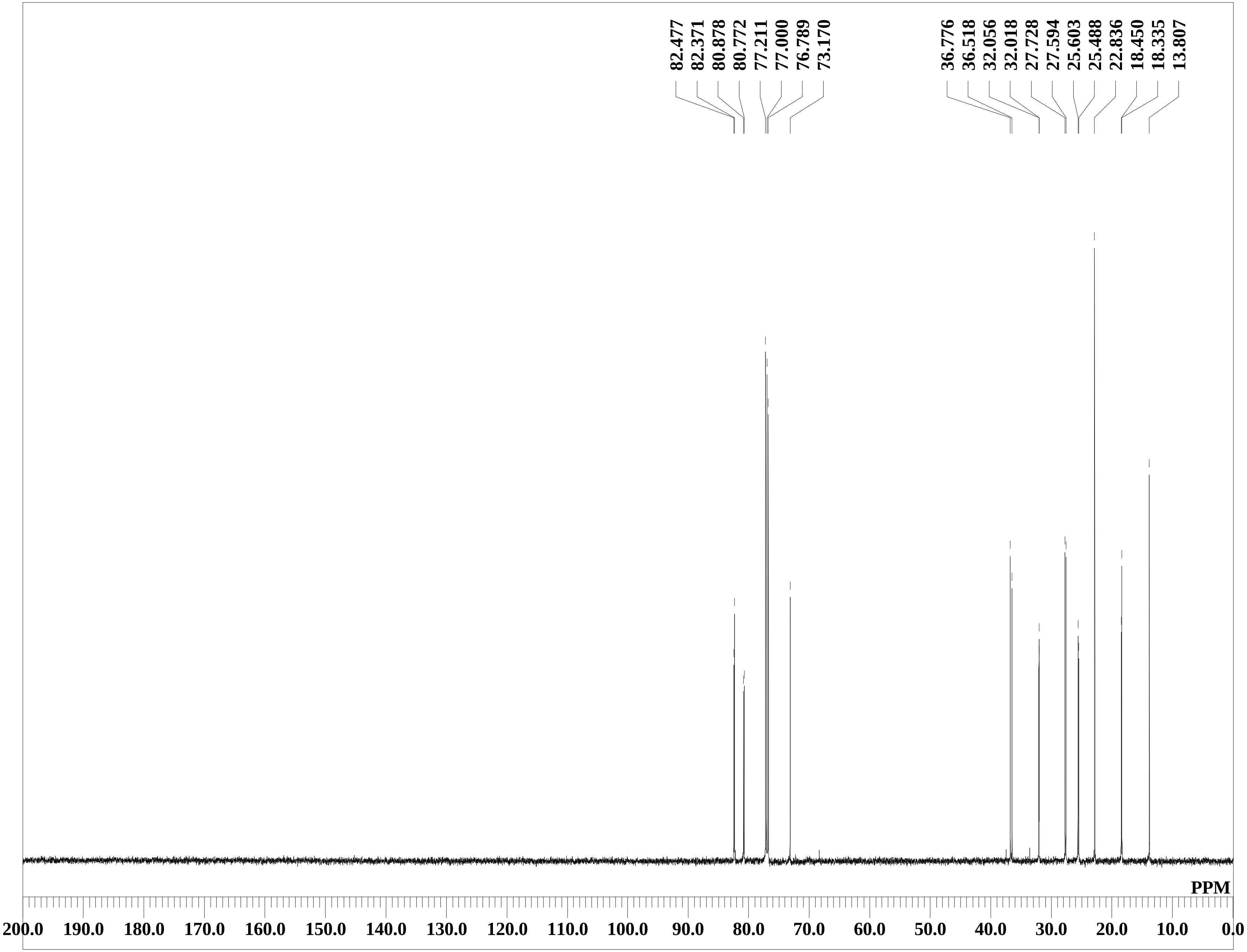
82.477
82.371
80.878
80.772
77.211
77.000
76.789
73.170
36.776
36.518
32.056
32.018
27.728
27.594
25.603
25.488
22.836
18.450
18.335
13.807
PPM
200.0 190.0 180.0 170.0 160.0 150.0 140.0 130.0 120.0 110.0 100.0 90.0 80.0 70.0 60.0 50.0 40.0 30.0 20.0 10.0 0.0

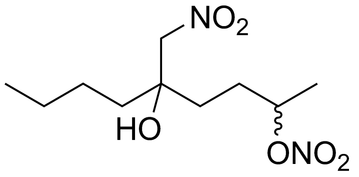
NO2
HO
ONO2

22

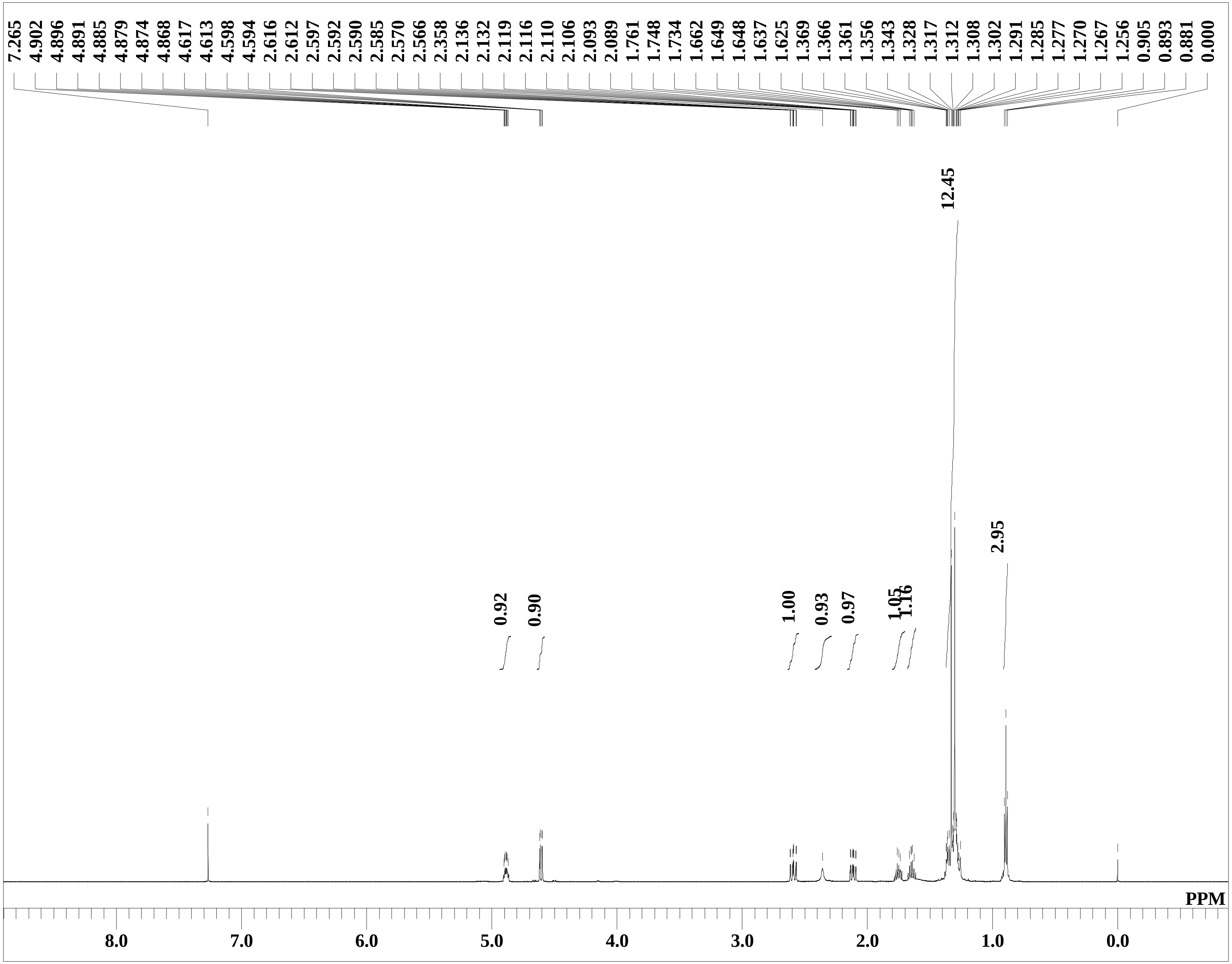
7.265
4.902
4.896
4.891
4.885
4.879
4.874
4.868
4.617
4.613
4.598
4.594
2.616
2.612
2.597
2.592
2.590
2.585
2.570
2.566
2.358
2.136
2.132
2.119
2.116
2.110
2.106
2.093
2.089
1.761
1.748
1.734
1.662
1.649
1.648
1.637
1.625
1.369
1.366
1.361
1.356
1.343
1.328
1.317
1.312
1.308
1.302
1.291
1.285
1.277
1.270
1.267
1.256
0.905
0.893
0.881
0.000
12.45
2.95
0.92
0.90
1.00
0.93
0.97
1.05
1.16
PPM
8.0
7.0
6.0
5.0
4.0
3.0
2.0
1.0
0.0

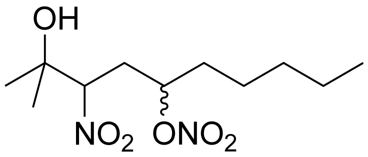
OH
NO2
ONO2

23

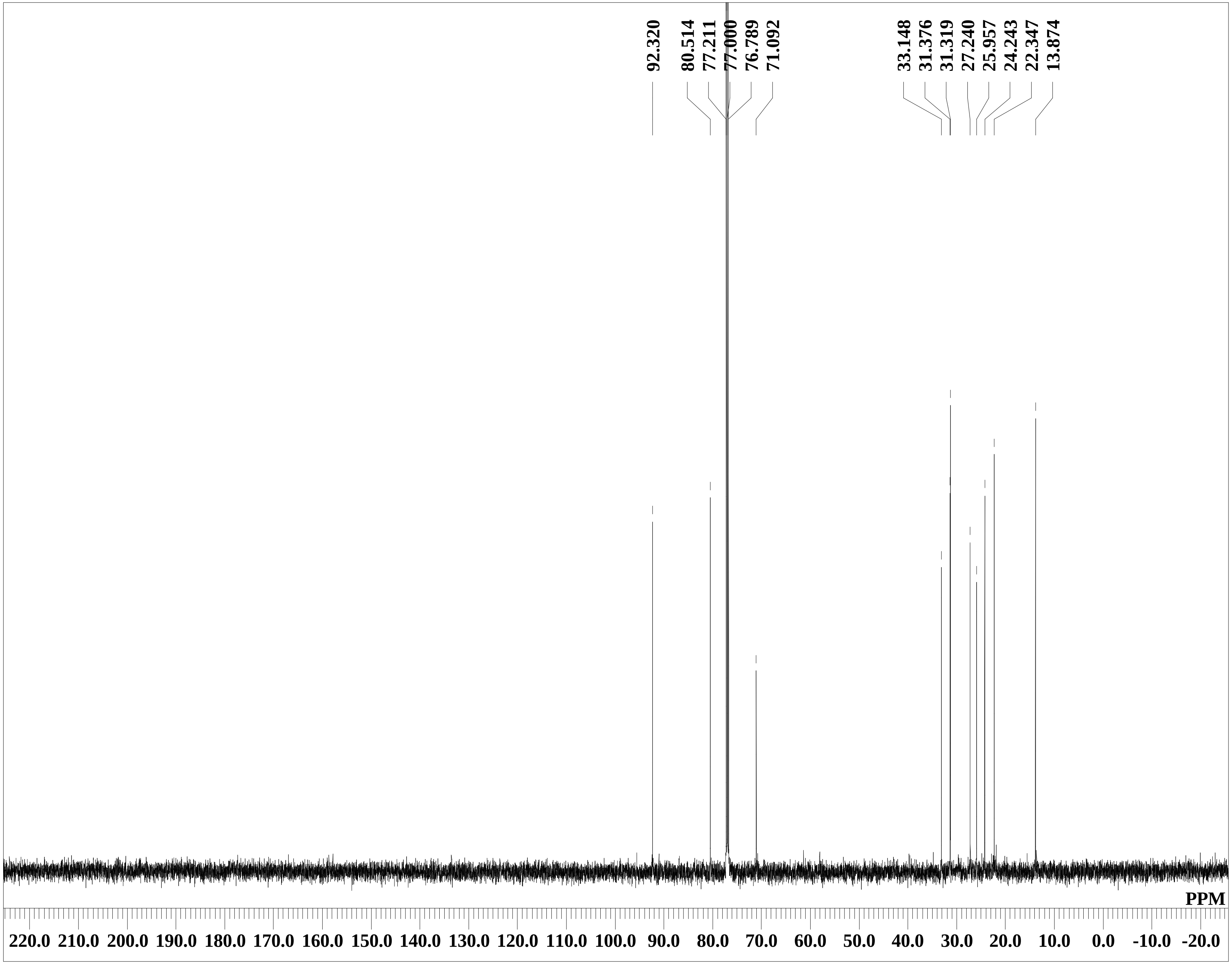
92.320
80.514
77.211
77.000
76.789
71.092
33.148
31.376
31.319
27.240
25.957
24.243
22.347
13.874
PPM
220.0 210.0 200.0 190.0 180.0 170.0 160.0 150.0 140.0 130.0 120.0 110.0 100.0 90.0 80.0 70.0 60.0 50.0 40.0 30.0 20.0 10.0 0.0 -10.0 -20.0

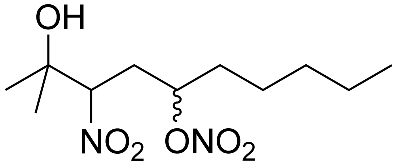
OH
NO_2
ONO_2

23

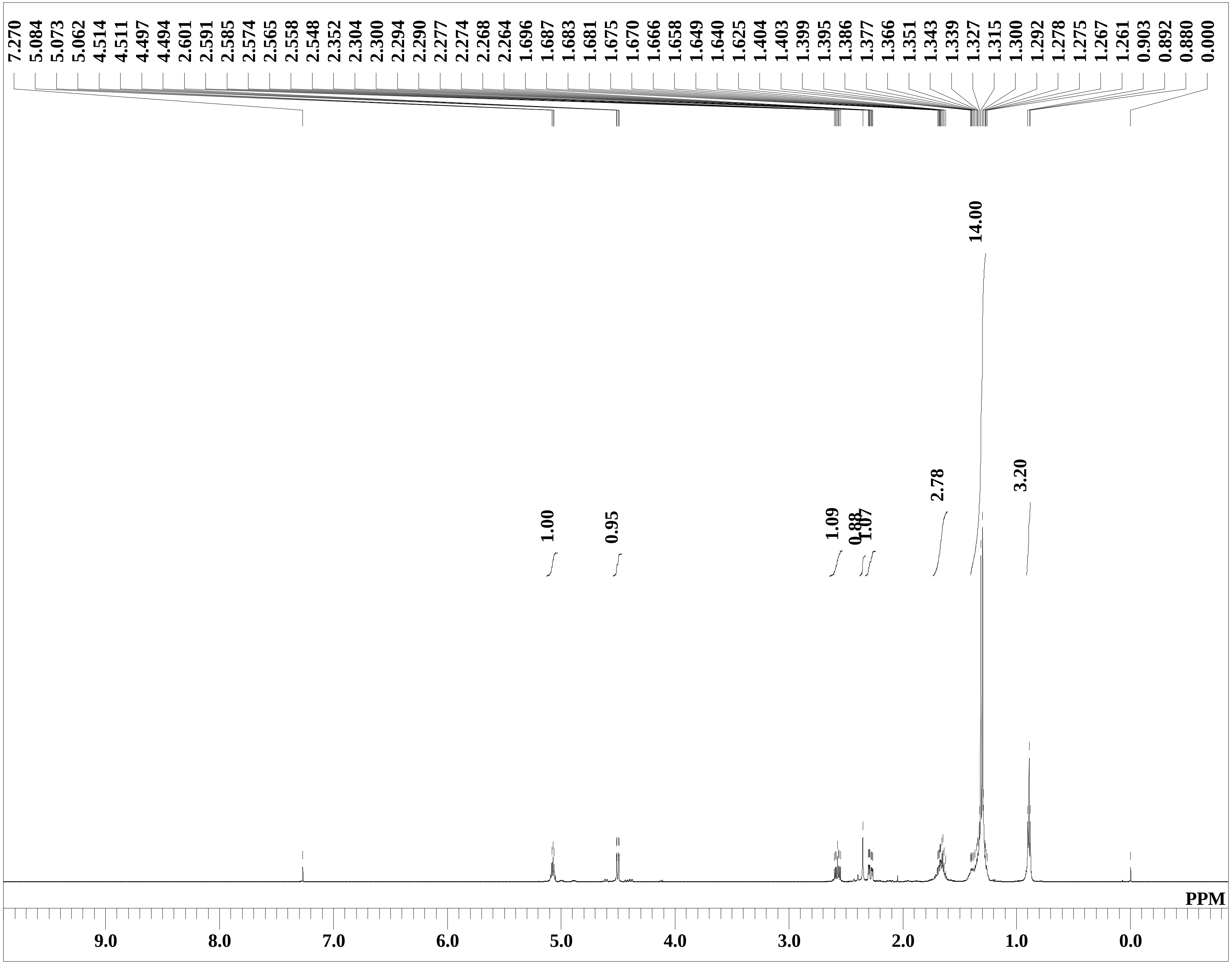

7.270 5.084 5.073 5.062 4.514 4.511 4.497 4.494 2.601 2.591 2.585 2.574 2.565 2.558 2.548 2.352 2.304 2.300 2.294 2.290 2.277 2.274 2.268 2.264 1.696 1.687 1.683 1.681 1.675 1.670 1.666 1.658 1.649 1.640 1.625 1.404 1.403 1.399 1.395 1.386 1.377 1.366 1.351 1.343 1.339 1.327 1.315 1.300 1.292 1.278 1.275 1.267 1.261 0.903 0.892 0.880 0.000
14.00
2.78
3.20
1.00
0.95
1.09
0.88
1.07
PPM
9.0
8.0
7.0
6.0
5.0
4.0
3.0
2.0
1.0
0.0

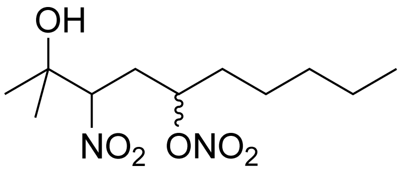

OH
NO2
ONO2

23'

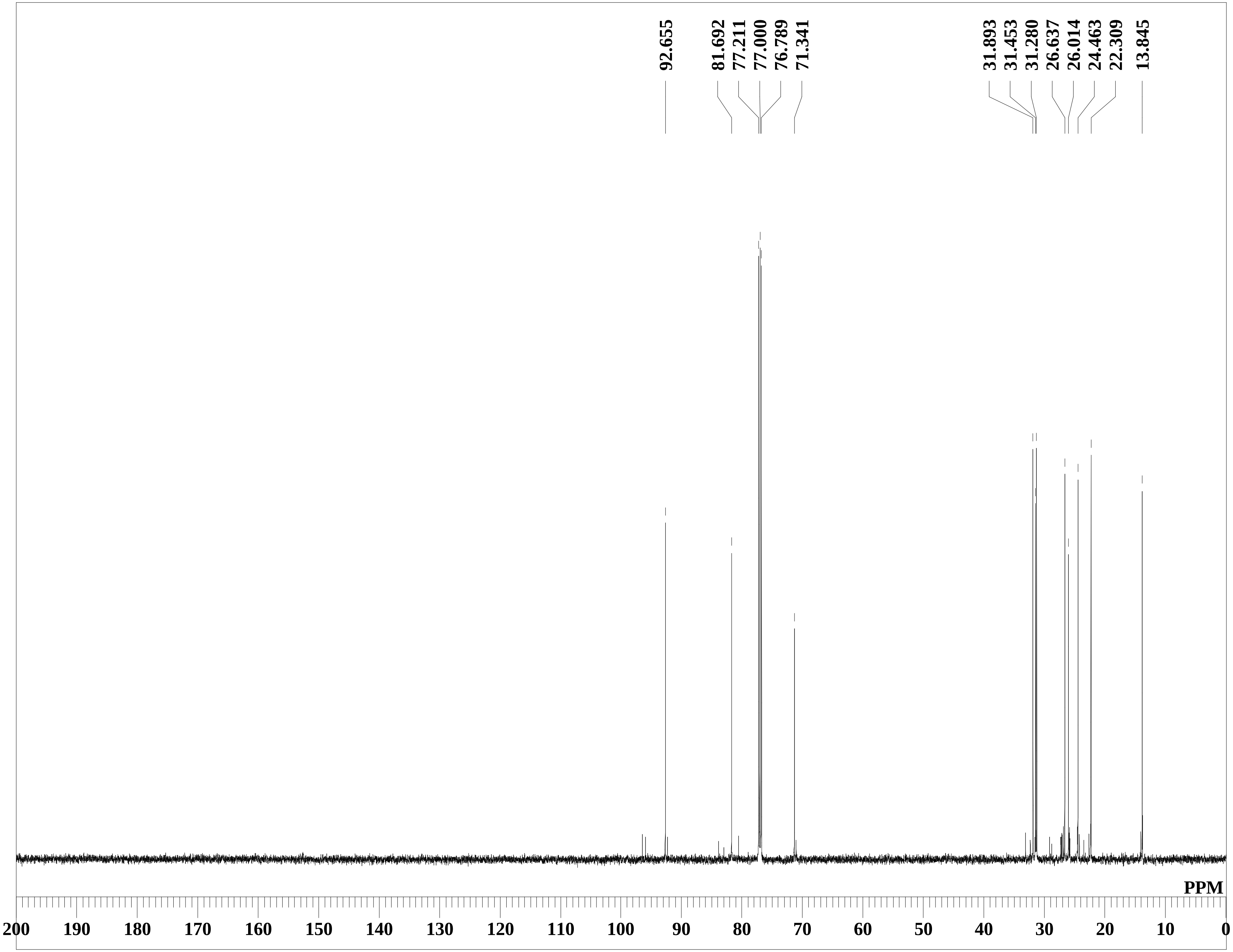
92.655
81.692
77.211
77.000
76.789
71.341
31.893
31.453
31.280
26.637
26.014
24.463
22.309
13.845
PPM
200 190 180 170 160 150 140 130 120 110 100 90 80 70 60 50 40 30 20 10 0

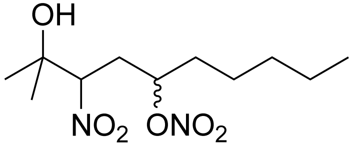
OH
NO2
ONO2

23'

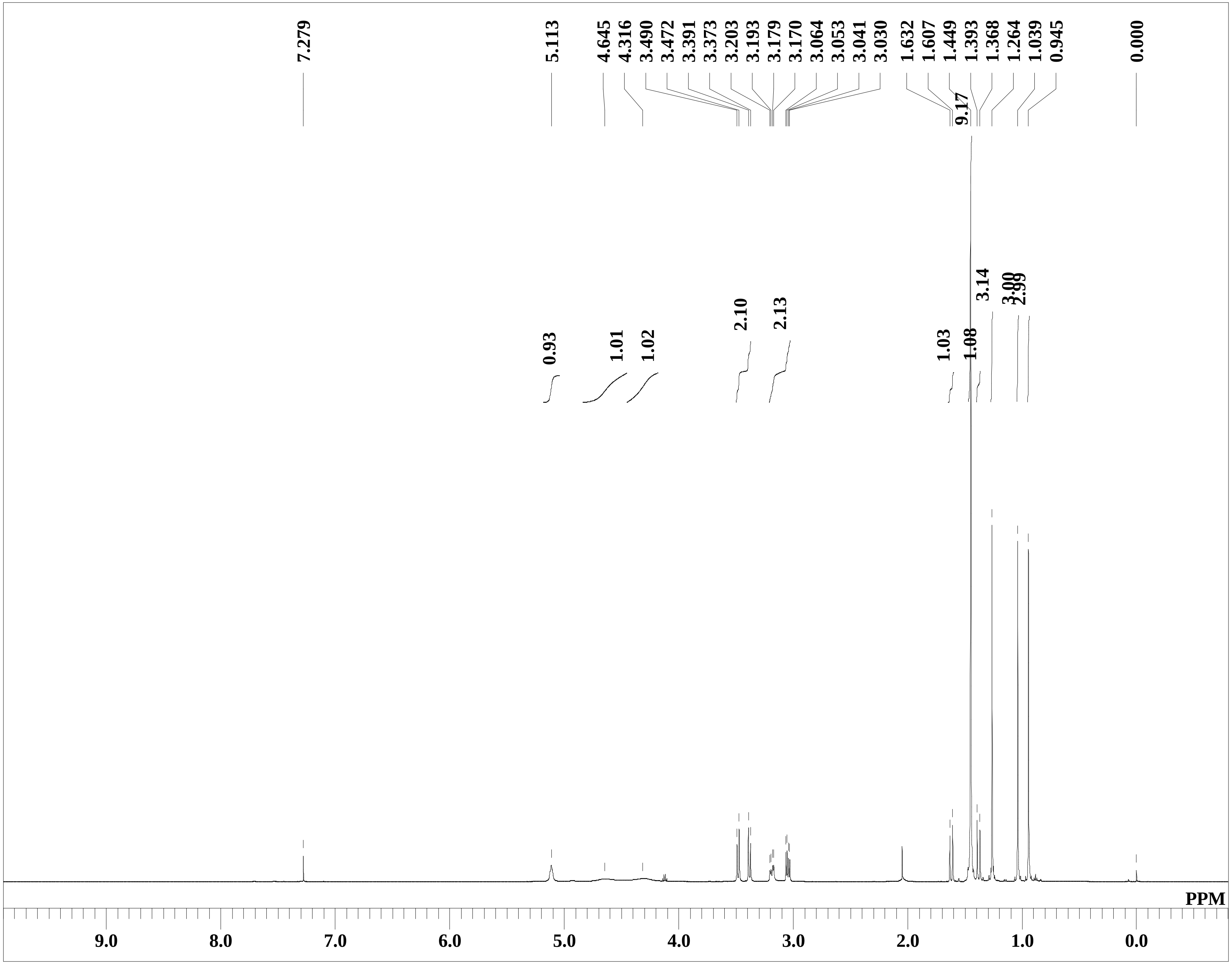
7.279
5.113
4.645
4.316
3.490
3.472
3.391
3.373
3.203
3.193
3.179
3.170
3.064
3.053
3.041
3.030
1.632
1.607
1.449
1.393
1.368
1.264
1.039
0.945
0.000
9.17
3.14
3.00
2.99
2.10
2.13
0.93
1.01
1.02
1.03
1.08
PPM
9.0
8.0
7.0
6.0
5.0
4.0
3.0
2.0
1.0
0.0

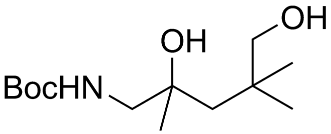
OH
OH
BocHN

25

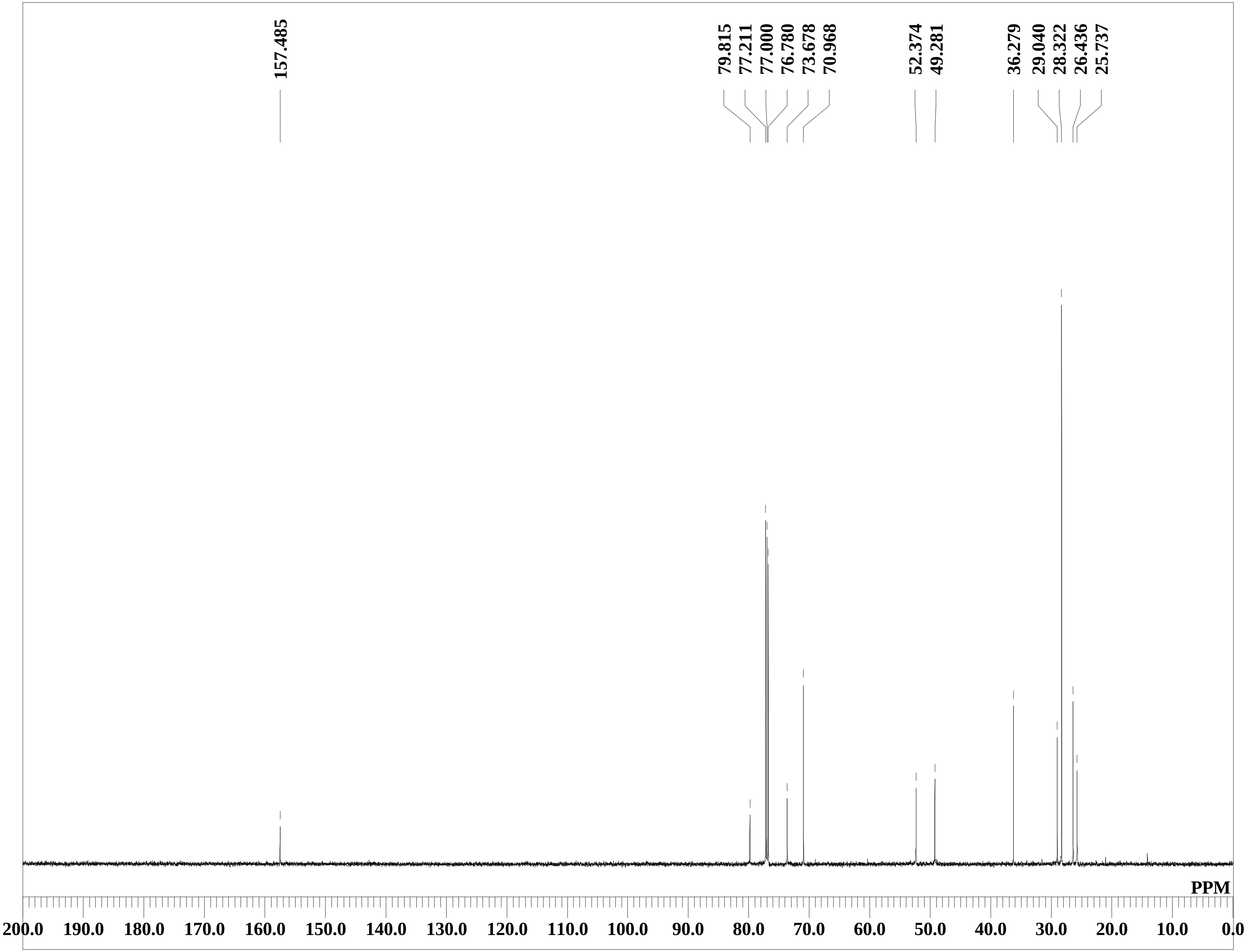
157.485
79.815
77.211
77.000
76.780
73.678
70.968
52.374
49.281
36.279
29.040
28.322
26.436
25.737
PPM
200.0
190.0
180.0
170.0
160.0
150.0
140.0
130.0
120.0
110.0
100.0
90.0
80.0
70.0
60.0
50.0
40.0
30.0
20.0
10.0
0.0

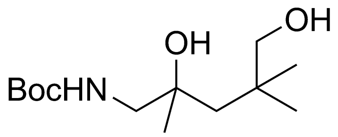
OH
OH
BocHN

25
